# Prenatal and progressive coenzyme Q_10_ administration to mitigate muscle dysfunction in mitochondrial disease

**DOI:** 10.1002/jcsm.13574

**Published:** 2024-10-02

**Authors:** Juan Diego Hernández‐Camacho, Cristina Vicente‐García, Lorena Ardila‐García, Ana Padilla‐Campos, Guillermo López‐Lluch, Carlos Santos‐Ocaña, Peter S. Zammit, Jaime J. Carvajal, Plácido Navas, Daniel J.M. Fernández‐Ayala

**Affiliations:** ^1^ Centro Andaluz de Biología del Desarrollo—CSIC Universidad Pablo de Olavide Seville Spain; ^2^ CIBERER Instituto de Salud Carlos III Madrid Spain; ^3^ Randall Centre for Cell and Molecular Biophysics King's College London London UK

**Keywords:** ageing, coenzyme Q, development, mitochondria, satellite cell, skeletal muscle

## Abstract

**Background:**

*ADCK* genes encode aarF domain‐containing mitochondrial kinases involved in coenzyme Q (CoQ) biosynthesis and regulation. Haploinsufficiency of ADCK2 in humans leads to adult‐onset physical incapacity with reduced mitochondrial CoQ levels in skeletal muscle, resulting in mitochondrial myopathy and alterations in fatty acid β‐oxidation. The sole current treatment for CoQ deficiencies is oral administration of CoQ_10_, which causes only partial recovery with postnatal treatment, underscoring the importance of early diagnosis for successful intervention.

**Methods:**

We used *Adck2* heterozygous mice to examine the influence of this gene on muscle structure, function and regeneration throughout development, growth and ageing. This investigation involved techniques including immunohistochemistry, analysis of CoQ levels, mitochondrial respiratory content, muscle transcriptome analysis and functional tests.

**Results:**

We demonstrated that *Adck2* heterozygous mice exhibit defects from embryonic development, particularly in skeletal muscle (1102 genes deregulated). *Adck2* heterozygous embryos were 7% smaller in size and displayed signs of delayed development. Prenatal administration of CoQ_10_ could mitigate these embryonic defects. Heterozygous *Adck2* mice also showed a decrease in myogenic cell differentiation, with more severe consequences in ‘aged’ mice (41.63% smaller) (*P* < 0.01). Consequently, heterozygous *Adck2* mice displayed accelerated muscle wasting associated with ageing in muscle structure (*P* < 0.05), muscle function (less grip strength capacity) (*P* < 0.001) and muscle mitochondrial respiration (*P* < 0.001). Furthermore, progressive CoQ_10_ administration conferred protective effects on mitochondrial function (*P* < 0.0001) and skeletal muscle (*P* < 0.05).

**Conclusions:**

Our work uncovered novel aspects of CoQ deficiencies, revealing defects during embryonic development in mammals for the first time. Additionally, we identified the gradual establishment and progression of the deleterious *Adck2* mouse phenotype. Importantly, CoQ_10_ supplementation demonstrated a protective effect when initiated during development.

## Introduction

Mitochondria are metabolic hubs and signalling platforms. They produce the bulk of adenosine triphosphate (ATP) by oxidative phosphorylation (OXPHOS) and are also essential for reactive oxygen species (ROS) production, signalling, cell death and inflammation.^1^, ^2^, ^3^ The OXPHOS system is formed by the electron transport chain (ETC) and the phosphorylation system, which comprises the integral membrane ATP synthase. The ETC includes four macromolecular complexes (I, II, III and IV) and mobile electron carriers as coenzyme Q (CoQ) and cytochrome *c* (Cyt *c*).^4^ CoQ is a lipid molecule found in all cell membranes and is conserved from proteobacteria to humans. CoQ is composed of a redox‐active fully substituted benzoquinone ring and a polyisoprenoid chain that maintains the molecule embedded into the lipidic bilayer.^5^ CoQ continuously goes through oxidation–reduction cycles, rotating between its oxidized (CoQ or ubiquinone) and reduced (CoQH_2_ or ubiquinol) forms.^6^ The canonical mitochondrial function of CoQ is electron transport in the ETC. CoQ carries electrons from complexes I (CI) and II (CII) to complex III (CIII), helping to create the electrochemical gradient that enables ATP synthesis by the ATP synthase (CV).^7^, ^8^ However, CoQ presents other crucial functions such as a structural component of CI and CIII.^9^, ^10^, ^11^ CoQ is also involved in the de novo synthesis of pyrimidines, β‐oxidation of fatty acids and branched‐chain amino acid oxidation, metabolism of proline and arginine, and detoxification of sulphide.^12^, ^13^, ^14^, ^15^

CoQ is mainly produced in the mitochondria by a group of nuclear‐encoded proteins (CoQ proteins), but the head and tail precursors are synthesized in the endoplasmic reticulum and cytosol.^16^ Inside mitochondria, serial modifications carried out by the CoQ proteins convert the precursors into functional CoQ molecules.^5^ CoQ deficiency groups heterogeneous syndromes, with a wide variety of clinical manifestations involving many tissues, organs and systems.^17^ Mutations in genes involved in CoQ biosynthesis or carrying out CoQ regulation functions produce CoQ deficiency.^5^, ^18^, ^19^ Several murine models have been developed to study diseases associated with CoQ deficiency. For example, the *Pdss2* deficiency mouse model exhibits autoimmune kidney disease^20^; mice lacking *Coq7* suffer hepatic impairment of respiratory chain function^21^; the *Coq8a* (*Adck3*) null model develops a slowly progressive cerebellar ataxia^22^; and *Coq9* knockouts display encephalopathy.^23^ These models provide insight into tissue‐specific damage that occurs in CoQ deficiency.

CoQ deficiency diagnosis in humans is based on CoQ levels, CI + CIII and CII + CIII activities, NADH, Cyt *c* and succinate–Cyt *c* reductase activities.^24^ However, when damage is usually detected, symptoms cannot be totally reversed.^25^ The initial phases of the molecular damage in this syndrome remain to be determined. Treatment is based on oral CoQ administration that improves CoQ deficiency symptoms, requiring early and appropriate supplementation.^26^, ^27^, ^28^ However, there are no data on the effects of long‐term CoQ administration in this syndrome, providing a rationale for the study of longitudinal CoQ supplementation in CoQ deficiency models.

*ADCK* genes encode members of the aarF domain‐containing mitochondrial kinases, which are a family of proteins involved in CoQ biosynthesis, regulation and maintenance. Mutations in *ADCK* genes are responsible for mitochondrial disorders.^29^ ADCK3 mutations produce autosomal recessive cerebellar ataxia,^30^ and ADCK4 mutations cause steroid‐resistant nephrotic syndrome.^31^ ADCK2 haploinsufficiency in humans produces adult‐onset myopathy with CoQ deficiency, accelerated physical incapacity and defective mitochondrial lipid metabolism. A heterozygous *Adck2* knockout model is able to recapitulate this mitochondrial syndrome.^32^ Metabolic and mitochondrial deregulation found in adult *Adck2* ^*+/−*^ mice resemble the physiology of old mice,^33^ making it also an attractive model to determine the impact of natural CoQ decrease during ageing, whose functional exploration is warranted.

In this study, we specifically investigated the appearance and progression of damage found in skeletal muscle from heterozygous *Adck2* mice. We also studied the impact of prenatal and longitudinal CoQ_10_ administration on the phenotype of mutant *Adck2* ^*+/−*^ mice. Unexpectedly, we discovered that ADCK2 depletion altered skeletal muscle formation during embryonic development. These alterations could be recapitulated during satellite cell differentiation in postnatal stages, and CoQ_10_ administration ameliorated this damage. Finally, we showed the progression of the deleterious phenotype in *Adck2* ^*+/−*^ mice with ageing and improvement by prenatal CoQ_10_ supplementation. Together, our data reveal the role of ADCK2 in mitochondria from skeletal muscle during embryonic development and ageing and provide insight into how prenatal CoQ_10_ administration can improve the phenotype of *Adck2* ^*+/−*^ mice.

## Materials and methods

### Mouse model, study approval and coenzyme Q_10_ administration

The *Adck2* knockout mouse model was generated in the C57BL/6J background, as previously described.^32^ All mouse studies were approved by the Universidad Pablo de Olavide Ethics Committee and carried out in accordance with the recommendations of Spanish and European guidelines. Reduced CoQ_10_ was administered to the mice in water (0.5 mg/mL) to achieve a final mean ingestion of 33.33–50 mg/kg/day per mouse (*Figure* *S1* *A*,*B*), in accordance with doses normally used in CoQ‐deficient patients. CoQ_10_ administration was initiated in adult mice and continued in their progeny for two generations to guarantee that mice were developed under CoQ_10_ administration. This produced a significant increase in plasma and in different tissues and a change in the CoQ_9_/CoQ_10_ ratio (*Figure* *S1* *B*) (please see the Methods section of the supporting information for further details).

### Microarray analysis

Transcriptome analyses were conducted at 17 days *post‐coitum* (17 d*pc*). Homogenization of the tissues was performed in TRIzol using a FastPrep‐24 5G homogenizer in Lysing Matrix D tubes. The RNA pellet was solubilized in RNase‐free water and stored at −80°C. RNA concentration and purity were determined spectrophotometrically in a Nanodrop, and RNA integrity was verified by electrophoresis in an agarose 1% (w/v) gel (*Figure* *S1* *C*). RNA was cleaned using the RNeasy MinElute Cleanup Kit (Qiagen) according to the manufacturer's instructions, and finally, RNA concentration was quantified in a Bioanalyzer. Samples were further processed at the Genomic Unit of CABIMER. A cRNA probe was synthesized and fragmented from each RNA sample using Affymetrix/Thermo Fisher protocols and kits provided by Qiagen. Each cRNA probe was then hybridized into independent Mouse Clarion D arrays. Hybridization and scanning of the arrays were performed according to the manufacturer's procedures. Computational analysis was undertaken using the Transcriptome Analysis Console (TAC) Software (Affymetrix/Thermo Fisher). Data were prefiltered according to their absolute fold change (higher than 1.75 at a linear scale) and their corrected false discovery rate (FDR) *P* value (*P* < 0.05).^34^ Transcriptomic data have been deposited at the National Center for Biotechnology Information (NCBI) Gene Expression Omnibus (GEO) database repository with the dataset identifier GSE253654.

### Skeletal muscle histology and immunochemistry

The tibialis anterior (TA) muscle was carefully dissected, embedded in Tissue‐Tek O.C.T.™ and immediately frozen in liquid nitrogen‐cooled isopentane. For fibre‐type composition, immunostaining was performed as previously described.^35^ For dystrophin immunostaining, slides were permeabilized with Triton X‐100, blocked for 1 h and incubated with primary antibodies overnight (OV). The next day, slides were washed and incubated with secondary antibodies for 2 h in darkness. Image acquisition was performed using a Stellaris Confocal Laser Scanning Microscope (10× objective), and analysis was performed with Fiji (ImageJ).^36^

### Primary satellite cell culture from mouse skeletal muscle and immunostaining

Satellite cells were isolated as previously described.^37^ Cells were fixed, washed and permeabilized with Triton X‐100/phosphate‐buffered saline (PBS). Cells were incubated with a blocking solution. Myosin heavy chain (MyHC) antibody (*Table* *S1*) was incubated OV. Cells were washed with PBS, and the secondary antibody (*Table* *S2*) was incubated for 2 h in darkness. Cells were washed with PBS and incubated with DAPI. Images were acquired on a Fluorescence Microscopy Zeiss Axio Imager M2 (10× objective). MyHC area and differentiation index were calculated using a high‐throughput image analysis R script.^38^

### Assessment of in vivo skeletal muscle regeneration

Barium chloride (BaCl_2_) (50 μL of 1.2% w/v in water) was injected into the right TA of mice to produce skeletal muscle damage and induce satellite cell‐mediated muscle regeneration.^39^ Next, 14‐day post‐injection (dpi) mice were sacrificed, and the TA was carefully dissected, frozen and immunolabelled as described above. Central myonuclei quantification was performed with Fiji (ImageJ) (included in *Table* *S4*).

An additional detailed description of the materials and methods can be found in the supporting information.

### Statistics

Data were expressed as mean ± SD. Data analyses were performed with the GraphPad Prism 9.0 software. When a comparison between two groups was performed, an unpaired two‐tailed *t* test was used. When more than two groups were studied, a one‐way (one parameter studied) or two‐way analysis of variance (ANOVA) (two or more parameters studied) with Tukey's multiple comparison post hoc tests was applied. *P* values <0.05 were considered statistically significant.

## Results

### Defects in *Adck2* ^*+/−*^ mice start during embryonic development

**Figure 1:**
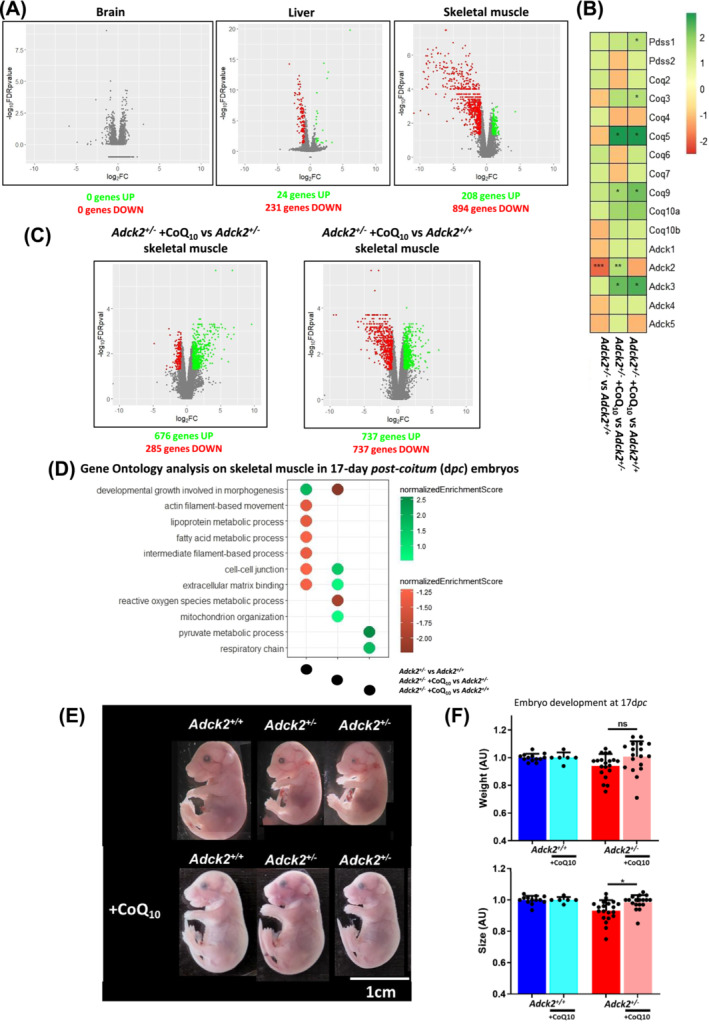
Embryonic developmental defects in heterozygous *Adck2* mice are rescued by CoQ_10_ administration. (A) Volcano plot representing the transcriptomic profile in brain, liver and skeletal muscle from 17‐day *post‐coitum* (d*pc*) embryos. Significant genes (false discovery rate [FDR] <0.05) with altered gene expression higher than 1.75‐fold are coloured in green if activated or in red if repressed. N = 3 per group. (B) CoQ pathway gene expression in skeletal muscle from embryos at 17 d*pc* on basal conditions and under CoQ_10_ prenatal administration. Activated gene expression is represented in green and repressed gene expression in red (comparisons represented: *Adck2* ^*+/−*^ vs. *Adck2* ^*+/+*^, *Adck2* ^*+/−*^ + CoQ_10_ vs. *Adck2* ^*+/−*^ and *Adck2* ^*+/−*^ + CoQ_10_ vs. *Adck2* ^*+/+*^). N = 3 per group. (C) Volcano plot representing the transcriptomic profile in skeletal muscle from *Adck2* ^*+/−*^ + CoQ_10_ versus *Adck2* ^*+/−*^ and from *Adck2* ^*+/−*^ + CoQ_10_ versus *Adck2* ^*+/+*^ of 17‐d*pc* embryos. Significant genes (FDR < 0.05) with an altered gene expression higher than 1.75‐fold are coloured in green if activated or in red if repressed. N = 3 per group. (D) Gene Ontology (GO) functional enrichment analysis on the genes differentially expressed in skeletal muscle from 17‐d*pc* embryos on basal conditions and under CoQ_10_ administration. Activated GO terms appear in green and repressed GO terms in red. N = 3 per group. (E) Representative images of *Adck2* ^*+/+*^ and *Adck2* ^*+/−*^ embryos at 17 d*pc* under with/without CoQ_10_ administration. (F) Normalized body weight of 17‐d*pc Adck2* ^*+/−*^ mutant embryos relative to *Adck2* ^*+/+*^ siblings of the same litter to reduce variability. Normalized length of embryos at 17‐d*pc Adck2* ^*+/−*^ mutant embryos relative to *Adck2* ^*+/+*^ siblings of the same litter to reduce variability (*Adck2* ^*+/+*^ N = 13; *Adck2* ^*+/+*^ + CoQ_10_ N = 6; *Adck2* ^*+/−*^ N = 20; and *Adck2* ^*+/−*^ + CoQ_10_ N = 18). ns, not significant. Data represent the mean ± SD. One‐way analysis of variance (ANOVA) test was applied. *P < 0.05, ^**^ *P* < 0.01 and ^***^ *P* < 0.001.

To evaluate whether damage observed in adult heterozygous *Adck2* ^*+/−*^ mice could originate during embryonic development, we explored the transcriptomic profile of three different tissues at 17 d*pc*: the brain, liver and skeletal muscle (*Figure* *1* *A*). Initially, no differentially expressed genes were found in the brain from embryos, whereas 255 genes were deregulated in the liver. In skeletal muscle, over 1102 genes were deregulated (*Figure* *1* *A*). Some of these transcriptional changes in skeletal muscle were confirmed at the protein level (*Figure* *S1* *F*). These differences in the transcriptomic profile, such as *Gpx3* (fold change: −38.65) or *Pkp1* (fold change: −85.61) in *Adck2* ^*+/−*^ versus *Adck2* ^*+/+*^, indicate that alterations in *Adck2* ^*+/−*^ mice start during embryonic development, and such changes are tissue‐specific, with the greatest deregulation in skeletal muscle, which is the main tissue affected in adult human patients.^32^

We also explored the transcriptomic profile in 17‐d*pc* embryos prenatally supplemented with CoQ_10_ (*Figure* *1* *B*). Specifically, the expression level of genes involved in CoQ biosynthesis and regulation was examined (*Figure* *1* *B*). As expected, the *Adck2* gene was significantly repressed in *Adck2* ^*+/−*^ embryos compared to *Adck2* ^*+/+*^. However, *Adck2* was upregulated in *Adck2* ^*+/−*^ embryos supplemented with CoQ_10_. CoQ_10_ administration also produced upregulation of *Pdss1*, *Coq3*, *Coq5*, *Coq9* and *Adck3* in *Adck2* ^*+/−*^ embryos. These alterations in gene expression within the CoQ biosynthesis pathway were induced by prenatal and chronic CoQ_10_ administration. Next, we examined the global gene expression of *Adck2* ^*+/−*^ embryos supplemented with CoQ_10_ versus *Adck2* ^*+/−*^ non‐supplemented and *Adck2* ^*+/+*^ (*Figure* *1* *C*), observing a huge impact of CoQ_10_ on gene expression. In agreement, gene ontologies (GOs) related to skeletal muscle formation and structure, such as ‘intermediate filament‐based process’ or ‘cell–cell junction’, were repressed in *Adck2* ^*+/−*^ embryos (*Figure* *1* *D*). When CoQ_10_ was administered to mutant embryos, expression of these GO terms was recovered to control levels, and new pathways involved in metabolism, including ‘mitochondrion organization’ or ‘pyruvate metabolic process’, were upregulated (*Figure* *1* *D*). We also evaluated the expression levels of genes involved in apoptosis, protein turnover, mitochondrial biogenesis, dynamics and mitophagy (*Figure* *S2A*–*D*). Under standard conditions, *Adck2* ^*+/−*^ showed few differences; particularly significant deregulation was found in genes related to caspase‐mediated cell death (*Figure* *S2A*). However, a modest upregulation was observed following CoQ_10_ administration. CoQ_10_ administration to control embryos (*Figure* *S2F*) also caused activation of metabolic and structural pathways. Some gene expression changes obtained from microarray studies were validated through quantitative reverse transcription polymerase chain reaction (RT‐qPCR) (*Figure* *S2G*). To assess if changes in gene expression associated with apoptosis and caspase‐mediated cell death (*Figure* *S3A*) could impact muscle during ageing, terminal deoxynucleotidyl transferase dUTP nick end labelling (TUNEL) staining was performed on TA muscle from old mice. A significant increase in the TUNEL‐positive cells/DAPI‐positive cells ratio was observed in *Adck2* ^*+/−*^ mice. However, this increase was not significant when CoQ_10_ was administered prenatally and longitudinally to the mice.

The gene expression changes observed could potentially affect normal embryonic development (*Figure* *1* *E*), while CoQ_10_ administration could modulate these changes. At 17 d*pc*, *Adck2* ^*+/−*^ embryos were smaller compared to control embryos (*Figure* *1* *F*), while CoQ_10_ administration increased and normalized their size (*Figure* *1* *F*).

### Haploinsufficiency of *Adck2* influences skeletal muscle structure and function through ageing

**Figure 2:**
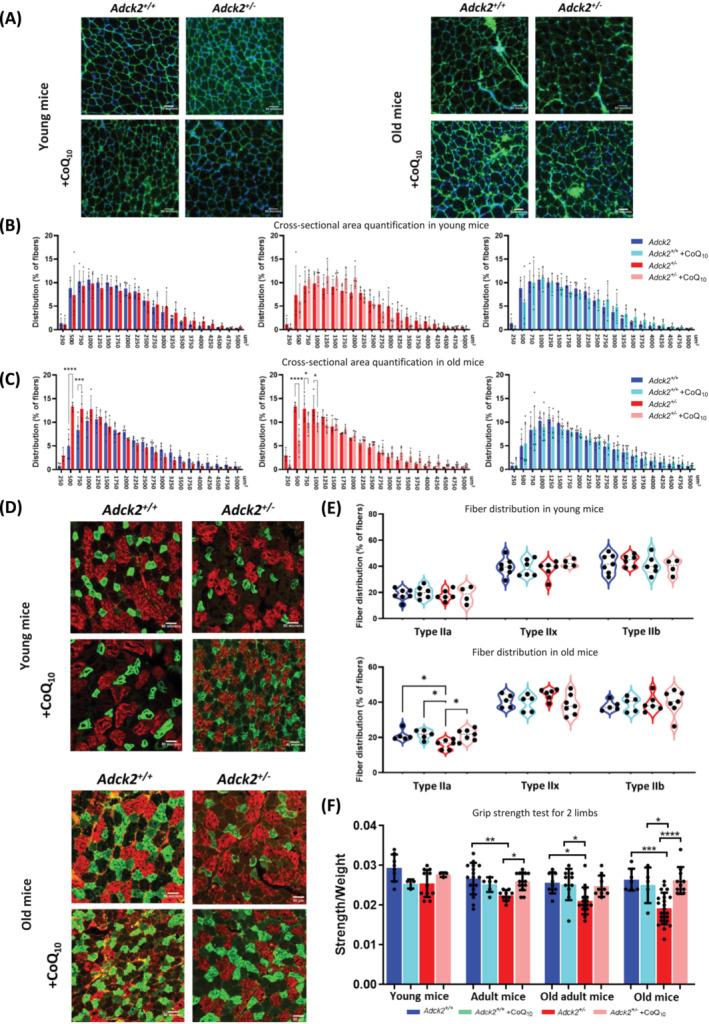
*Adck2* haploinsufficiency affects skeletal muscle structure and function during ageing. (A) Representative images of tibialis anterior (TA) muscles from young and old mice with/without CoQ_10_ administration. Dystrophin immunolabelling (green) and a nuclear DAPI counterstain (blue). Scale bar 50 μm. (B) Cross‐sectional area (CSA) quantification for the complete transversal section of TA muscle in young mice. Distribution of myofibres in relation to CSA. The *x* axis represents the area, and the *y* axis represents the percentage of the muscle myofibres in each category (*Adck2* ^*+/+*^ N = 4; *Adck2* ^*+/+*^ + CoQ_10_ N = 4; *Adck2* ^*+/−*^ N = 4; and *Adck2* ^*+/−*^ + CoQ_10_ N = 4). (C) CSA quantification for the complete transversal section of TA muscle in old mice. Distribution of myofibres in relation to CSA. The *x* axis represents the area, and the *y* axis represents the percentage of the muscle myofibres in each category (*Adck2* ^*+/+*^ N = 4; *Adck2* ^*+/+*^ + CoQ_10_ N = 4; *Adck2* ^*+/−*^ N = 4; and *Adck2* ^*+/−*^ + CoQ_10_ N = 3). (D) Representative images of TA muscles from young (upper panel) and old mice (bottom panel) with/without CoQ_10_ administration immunolabelled for Type IIa (green), IIb (red) and IIx myofibres (unlabelled). Scale bar 50 μm. (E) Myofibre composition analysis (young mice [*Adck2* ^*+/+*^ N = 7; *Adck2* ^*+/+*^ + CoQ_10_ N = 6; *Adck2* ^*+/−*^ N = 6; and *Adck2* ^*+/−*^ + CoQ_10_ N = 4]; old mice [*Adck2* ^*+/+*^ N = 5; *Adck2* ^*+/+*^ + CoQ_10_ N = 5; *Adck2* ^*+/−*^ N = 6; and *Adck2* ^*+/−*^ + CoQ_10_ N = 7]). (F) Results for grip strength test for two limbs normalized by body weight (young mice [*Adck2* ^*+/+*^ N = 8; *Adck2* ^*+/+*^ + CoQ_10_ N = 3; *Adck2* ^*+/−*^ N = 11; and *Adck2* ^*+/−*^ + CoQ_10_ N = 3]; adult mice [*Adck2* ^*+/+*^ N = 16; *Adck2* ^*+/+*^ + CoQ_10_ N = 6; *Adck2* ^*+/−*^ N = 9; and *Adck2* ^*+/−*^ + CoQ_10_ N = 15]; old adult mice [*Adck2* ^*+/+*^ N = 8; *Adck2* ^*+/+*^ + CoQ_10_ N = 10; *Adck2* ^*+/−*^ N = 15; and *Adck2* ^*+/−*^ + CoQ_10_ N = 9]; and old mice [*Adck2* ^*+/+*^ N = 7; *Adck2* ^*+/+*^ + CoQ_10_ N = 5; *Adck2* ^*+/−*^: N = 23; and *Adck2* ^*+/−*^ + CoQ_10_ N = 10]). Data represent the mean ± SD. One‐way or two‐way analysis of variance (ANOVA) test was applied. *P < 0.05, ^**^ *P* < 0.01, ^***^ *P* < 0.001 and ^****^ *P* < 0.0001.

To assess if these changes detected in the transcriptomic profile from *Adck2* ^*+/−*^ embryos persist later in life, we evaluated the general appearance of the mice as they aged (*Figure* *S3B*) and measured tibial bone length to assess body development (*Figure* *S3C*). We also investigated if defects in prenatal myogenesis in *Adck2* ^*+/−*^ mice had a negative impact on skeletal muscle function/structure, which will also provide insight into CoQ_10_ disorder in human.^32^ We examined the myofibre size and composition of the TA in adult 3‐month‐old and aged 2‐year‐old mice (*Figures* *2* *A*, *S3D*,*E* and *S4A*,*B*).

First, the size of all myofibres from the entire transverse section of the TA muscle was evaluated and classified into different intervals to assess the distribution of the myofibre population (*Figures* *2* *B*,*C*, *S3D*,*E* and *S5A*,*B*). Young mice did not show any differences in myofibre cross‐sectional area (CSA) (*Figures* *2* *B* and *S3D*) or minimal Feret's diameter (*Figure* *S5A*). However, myofibres from old *Adck2* ^*+/−*^ mice showed a decrease in size associated with ageing (*Figures* *2* *C*, *S3E* and *S5B*). In contrast, older *Adck2* ^*+/−*^ mice, when administered CoQ_10_, exhibited a lower proportion of small myofibres. This suggests that progressive CoQ_10_ supplementation counteracted this defect (*Figures* *2* *C*, *S3E* and *S5B*). Regarding myofibre‐type composition (*Figures* *2* *D* and *S4C*,*D*), no differences were observed in young mice (*Figure* *2* *E*). However, old *Adck2* ^*+/−*^ mice showed a lower proportion of Type IIa myofibres compared to *Adck2* ^*+/+*^, while CoQ_10_ administration rescued this phenotype to *Adck2* ^*+/+*^ levels (*Figure* *2* *E*).

These results indicate that haploinsufficiency in *Adck2* ^*+/−*^ mice affects skeletal muscle structure and formation. Next, we examined muscle physical capacity through ageing. Physical capacity was assessed with two‐ and four‐limb grip strength tests and a weight lifting test (*Figures* *2* *F* and *S5C*,*D* and *Table* *S5*). We observed a progressive decrease in grip strength capacity associated with ageing, which was more pronounced in *Adck2* ^*+/−*^ compared to *Adck2* ^*+/+*^ mice. However, CoQ_10_ administration at each age restored the grip strength capacity to levels found in *Adck2* ^*+/+*^ of the same age. This phenotype was not attributed to changes in body mass (*Figure* *S5C*–*F*) or to body growth as assessed by tibial length (*Figure* *S3C*). In parallel, the oxidative metabolism‐dependent pathway was examined by voluntary running wheel activity over 3 days in a TSE PhenoMaster system through ageing (*Figure* *S6A*–*D*). *Adck2* ^*+/−*^ showed a greater decrease in voluntary running performance as assessed by distance recorded in the course of ageing compared to *Adck2* ^*+/+*^ mice. However, *Adck2* ^*+/−*^ supplemented with CoQ_10_ showed a smaller decrease in freely running distance (*Figure* *S6A*–*D*), reinforcing the idea that CoQ_10_ could preserve skeletal muscle health in mutant mice through ageing. In relation to muscle wasting and loss of physical capacity observed in *Adck2* ^*+/−*^ mice, we assessed mitochondrial content, dynamics and mitophagy markers but found no significant differences in relevant proteins (*Figures* *4* *E* and *S7A*,*B*). These results suggest that the muscle wasting observed in aged mice was not associated with a reduction in mitochondrial content.

### *Adck2* haploinsufficiency impacts myogenesis in young mice

**Figure 3:**
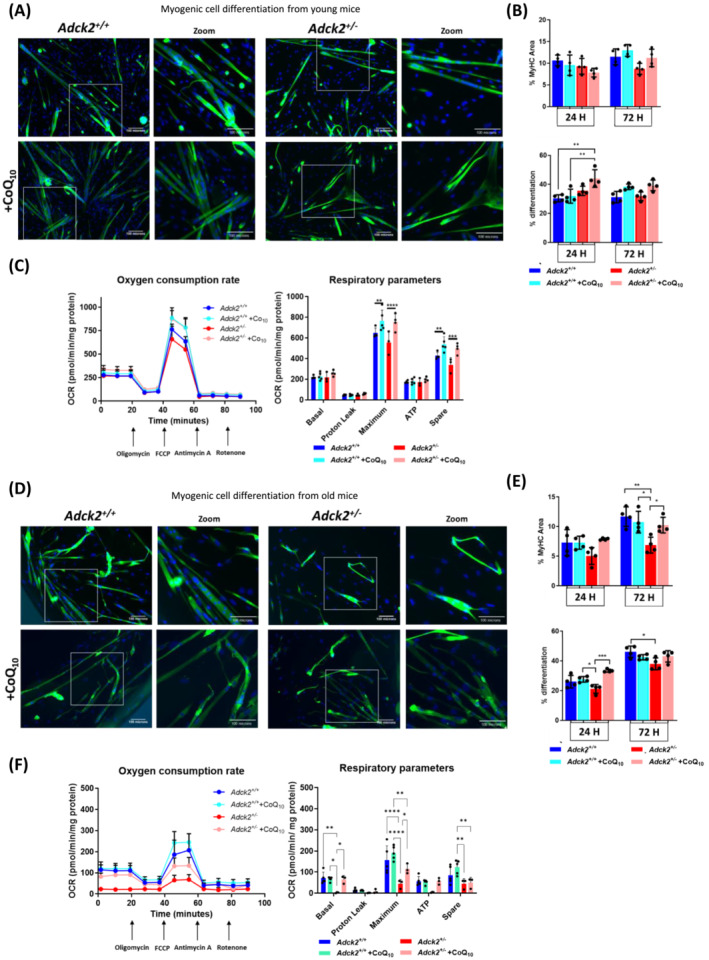
Impact of *Adck2* haploinsufficiency on myogenic differentiation of satellite cells. (A) Representative images of young mice satellite cell‐derived myotubes 72 h after induction with/without CoQ_10_ administration immunolabelled for myosin heavy chain (MyHC) (green) and nuclei counterstained with DAPI (blue). Scale bar 100 μm. (B) MyHC area and differentiation index of myotubes from young mice. (C) Oxygen consumption rate in myotubes from young mice. One representative experiment of three independent experiments is shown. (D) Representative images of old mice satellite cell‐derived myotubes 72 h after induction with/without CoQ_10_ administration immunolabelled for MyHC (green) and nuclei counterstained with DAPI (blue). Scale bar 100 μm. (E) MyHC area and differentiation index of myotubes from old mice. (F) Oxygen consumption rate in myotubes from old mice. One representative experiment of three independent experiments is shown. Respirometric assays were performed 72 h after induction of differentiation. Data represent the mean ± SD. One‐way analysis of variance (ANOVA) test was applied. *P < 0.05, ^**^ *P* < 0.01, ^***^ *P* < 0.001 and ^****^ *P* < 0.0001.

To determine whether changes observed in skeletal muscle could impact myogenesis in *Adck2* ^*+/−*^ mice, we examined this process through adult resident muscle stem cells, known as satellite cells, which are essential for skeletal muscle growth, repair and remodelling. Adult muscle stem cells were isolated from the extensor digitorum longus (EDL) muscle of 3‐month‐old *Adck2* ^*+/+*^ and *Adck2* ^*+/−*^ mice in basal conditions and under CoQ_10_ administration (*Figure* *3* *A*). Cells from mice under CoQ_10_ administration in vivo were also supplemented with CoQ_10_ in vitro. Differentiation was evaluated by measuring myotube growth and MyHC area at 24 and 72 h (*Figure* *3* *B*). Myotubes from young *Adck2* ^*+/−*^ mice did not exhibit any differences in MyHC area compared to the other groups. However, CoQ_10_ significantly boosted the differentiation index in mutant myotubes at 24 h (*Figure* *3* *B*).

Given the significance of oxidative metabolism in myogenic cell differentiation, we next examined mitochondrial activity. *Adck2* ^*+/−*^ myotubes at 72 h displayed lower oxygen consumption than *Adck2* ^*+/+*^ myotubes when glucose (maximal and spare respiration) (*Figure* *3* *C*) was used as a substrate. Both *Adck2* ^*+/+*^ and *Adck2* ^*+/−*^ myotubes supplemented with CoQ_10_ showed an increase in maximum oxygen consumption rate in respirometry assays with glucose substrate (*Figure* *3* *C*), indicating an improvement of respiration in vitro by CoQ_10_ administration.

### *Adck2* haploinsufficiency impacts myogenesis and mitochondrial function in old mice

To investigate the effect of ageing on satellite cell differentiation, we evaluated adult muscle stem cells isolated from the EDL muscles of 2‐year‐old mice on basal conditions and under longitudinal CoQ_10_ administration (*Figure* *3* *D*). *Adck2* ^*+/−*^ cells exhibited defective myogenic differentiation, as indicated by the reduced MyHC area at 72 h (*Figure* *3* *E*). The percentage of differentiated cells, evaluated by the ratio of nuclei in cells expressing MyHC to the total nuclei, was also reduced in *Adck2* ^*+/−*^ at 72 h, indicating impaired differentiation capacity. CoQ_10_ administration increased the differentiation capacity of mutant myotubes with respect to the MyHC area (*Figure* *3* *E*).

*Adck2* ^*+/−*^ myotubes from 2‐year‐old mice showed significantly compromised basal and maximum oxygen consumption compared to *Adck2* ^*+/+*^ myotubes when glucose was used as a substrate (*Figure* *3* *F*). However, supplementation of *Adck2* ^*+/−*^ myotubes with CoQ_10_ led to increased oxygen consumption rates (*Figure* *3* *F*). In summary, *Adck2* ^*+/−*^ mice exhibited compromised postnatal myogenesis characterized by reduced mitochondrial respiration. This defective capacity of *Adck2* ^*+/−*^ mice was significantly exacerbated in older mice. Given the improvement in mitochondrial respiration associated with CoQ_10_ administration, we next evaluated CoQ_10_ impact on mitochondrial morphology in myogenic cells. We labelled the mitochondria of both WT and *Adck2* ^*+/−*^ myogenic cells, with and without CoQ_10_ administration. We found an increase in mitochondrial area, perimeter and Feret diameter, along with a decrease in circularity, suggesting that CoQ_10_ administration was associated with mitochondrial elongation in myogenic cells (*Figure* *S7C*,*D*).

### Coenzyme Q_10_ administration ameliorates the bioenergetic decline in *Adck2* ^*+/−*^ mice

**Figure 4:**
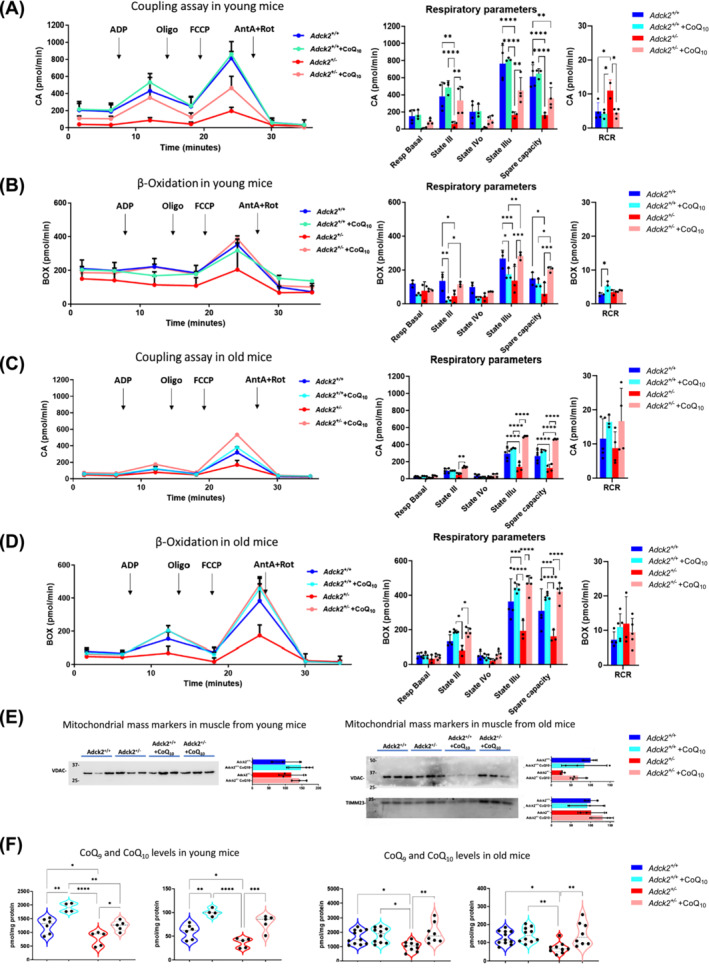
Mitochondrial bioenergetic decline in *Adck2* mice associated with ageing. (A) Coupling assay and (B) fatty acid β‐oxidation in mitochondria isolated from the skeletal muscle of young mice. For each experiment, 10 μg of mitochondrial suspension was seeded per well. One representative experiment of three independent experiments is shown. (C) Coupling assay and (D) fatty acid β‐oxidation in mitochondria isolated from the skeletal muscle of old mice. For each experiment, 10 μg of mitochondrial suspension was seeded per well. One representative experiment of three independent experiments is shown. (E) Protein markers of mitochondrial mass in skeletal muscle from young and old mice (young mice [*Adck2* ^*+/+*^ N = 3; *Adck2* ^*+/+*^ + CoQ_10_ N = 3; *Adck2* ^*+/−*^ N = 3; and *Adck2* ^*+/−*^ + CoQ_10_ N = 3]; old mice [*Adck2* ^*+/+*^ N= 3; *Adck2* ^*+/+*^ + CoQ_10_ N = 3; *Adck2* ^*+/−*^ N = 3; and *Adck2* ^*+/−*^ + CoQ_10_ N = 3]). (F) CoQ_9_ and CoQ_10_ levels in mitochondria isolated from skeletal muscle of young mice (*Adck2* ^*+/+*^ N = 6; *Adck2* ^*+/+*^ CoQ_10_ N = 4; *Adck2* ^*+/−*^ N = 5; and *Adck2* ^*+/−*^ CoQ_10_ N = 5). CoQ_9_ and CoQ_10_ levels in mitochondria isolated from skeletal muscle of old mice (*Adck2* ^*+/+*^ N = 11; *Adck2* ^*+/+*^ + CoQ_10_ N = 10; *Adck2* ^*+/−*^ N = 9; and *Adck2* ^*+/−*^ + CoQ_10_ N = 7). RCR, respiratory control ratio. Data represent the mean ± SD. One‐way analysis of variance (ANOVA) test was applied. *P < 0.05, ^**^ *P* < 0.01, ^***^ *P* < 0.001 and ^****^ *P* < 0.0001.

Based on these results obtained in the characterization of skeletal muscle during ageing, we found that mitochondrial function in *Adck2* ^*+/−*^ mice was compromised. To gain further insight, we performed respirometry analyses in isolated skeletal muscle mitochondria using either pyruvate/malate to stimulate the Krebs cycle and CI‐directed respiration or palmitoyl‐l‐carnitine/malate to stimulate fatty acid β‐oxidation (*Figure* *4* *A*–*D*).

Mitochondria from young and old *Adck2* ^*+/−*^ mice had compromised oxygen consumption characterized by a lower State III and State IIIu respiration status (*Figure* *4* *A*,*C*). State III represents maximal respiration in a coupled state, while State IIIu illustrates the mitochondria's maximum respiratory capacity in an uncoupled state. Additionally, skeletal muscle mitochondria from young and old *Adck2* ^*+/−*^ mice also presented lower oxygen consumption during β‐oxidation assays (*Figure* *4* *B*,*D*), indicating compromised mitochondrial function independent of ageing and regardless of substrate. However, skeletal muscle mitochondria from *Adck2* ^*+/−*^ mice supplemented with CoQ_10_ exhibited an increase in oxygen consumption in coupling and β‐oxidation assays in young and old mice. CoQ_10_ administration induces higher substrate efficiency while activating the different bioenergetic pathways involved in mitochondrial respiration. These results are consistent with the respirometry assays conducted in myotubes ex vivo, where myotubes derived from *Adck2* ^*+/−*^ mice exhibited lower respiration, and CoQ_10_ supplementation resulted in an increase in respiration. Mitochondrial mass markers in skeletal muscle from old mice did not exhibit decreased content, suggesting that the reduction in mitochondrial respiration was not associated with a decrease in mitochondrial mass (*Figures* *4* *E* and *S7A*).

We proceeded to analyse the assembly of OXPHOS complexes to explore whether the alterations identified in mitochondrial respiration could be attributed to modifications in the structure of the complexes. The compromised mitochondrial respiration observed in *Adck2* ^*+/−*^ mice was not directly correlated with an inefficient assembly of OXPHOS complexes (*Figure* *S8A*–*E*). Instead, we tested whether changes in mitochondrial respiration detected in the skeletal muscle of *Adck2* ^*+/−*^ mice may be influenced by the mitochondrial levels of CoQ.

Indeed, when we analysed the CoQ_9_ and CoQ_10_ in isolated mitochondria from both genotypes on basal conditions and under CoQ_10_ administration (*Figures* *4* *F* and *S8F*), we found that skeletal muscle mitochondria from young *Adck2* ^*+/−*^ mice showed significantly lower levels of CoQ_9_ and CoQ_10_ in mitochondria, a reduction that was also observed in old *Adck2* ^*+/−*^ mice. CoQ_10_ administration increased mitochondrial CoQ_9_ and CoQ_10_ pools in both young and old *Adck2* ^*+/−*^ mice. Overall, *Adck2* ^*+/−*^ mice showed a decrease in mitochondrial CoQ_9_ and CoQ_10_ levels and *Adck2* expression level (*Figure* *S8G*). We hypothesize that this reduction compromised mitochondrial respiration from early life and so resulted in skeletal muscle remodelling through ageing. However, CoQ_10_ prenatal and adulthood supplementation stabilized CoQ_9_ and CoQ_10_ levels in skeletal muscle mitochondria and maintained mitochondrial function, protecting skeletal muscle from more serious deleterious effects during ageing.

### Ablation of *Adck2* hinders the muscle's ability to respond to in vivo muscle damage

**Figure 5:**
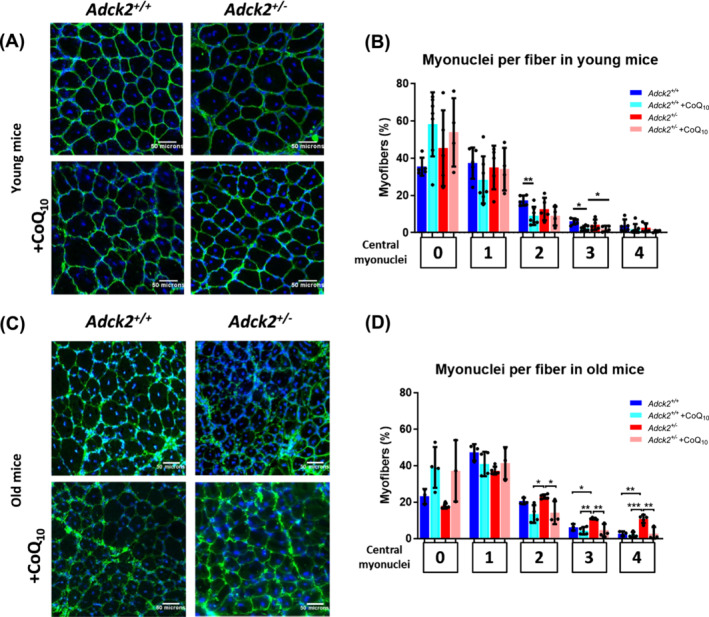
Impact of *Adck2* in skeletal muscle regeneration in young and old mice. (A) Representative images of transversal sections of tibialis anterior (TA) muscle 14 days post‐injection (dpi) in young mice with/without CoQ_10_ administration immunolabelled for dystrophin to delimit the myofibres (green) with a DAPI nuclear counterstain (blue). Scale bar 50 μm. (B) Distribution of myofibres according to the number of central myonuclei in young mice (*Adck2* ^*+/+*^ N = 5; *Adck2* ^*+/+*^ + CoQ_10_ N = 7; *Adck2* ^*+/−*^ N = 5; and *Adck2* ^*+/−*^ + CoQ_10_ N = 4). (C) Representative images of transversal sections of TA muscle 14 dpi in old mice with/without CoQ_10_ administration immunolabelled for dystrophin to delimit the myofibres (green) with a DAPI nuclear counterstain (blue). Scale bar 50 μm. (D) Distribution of myofibres according to the number of central myonuclei in old mice (*Adck2* ^*+/+*^ N = 3; *Adck2* ^*+/+*^ + CoQ_10_ N = 4; *Adck2* ^*+/−*^ N = 5; and *Adck2* ^*+/−*^ + CoQ_10_ N = 3). Data represent the mean ± SD. One‐way analysis of variance (ANOVA) test was applied. *P < 0.05, ^**^ *P* < 0.01 and ^***^ *P* < 0.001.

Given the observed impairment in satellite cell differentiation and increased muscle wasting in *Adck2* ^*+/−*^ mice, we subjected skeletal muscle to in vivo damage. To achieve this, we injected BaCl_2_ into the TA muscle of 3‐month‐old mice to induce skeletal muscle damage. Subsequently, we evaluated the regenerative capacity at 14 dpi (*Figure* *5* *A*,*B*). The longitudinal alignment of the nuclei on the centre of the myofibre was quantified for each individual myofibre from the entire transversal section of the TA muscle (*Figure* *S9A*,*B*) as an indicator of regenerated muscle. CoQ_10_ administration tended to reduce the number of central nuclei in myofibres, which are associated with skeletal muscle regeneration (*Figure* *5* *B*). However, no differences were found between *Adck2* ^*+/−*^ and WT mice.

We also examined the skeletal muscle regeneration capacity in response to in vivo muscle damage in old mice (*Figure* *5* *C*). Again, BaCl_2_ was injected into the TA muscle to produce skeletal muscle damage. The number of central nuclei in myofibres was assessed 14 days after BaCl_2_ injection in both groups of mice supplemented with/without CoQ_10_ (*Figure* *S9B*). There was a noticeable trend towards a higher number of myofibres with zero central myonuclei in mice supplemented with CoQ_10_, suggesting a potentially enhanced defence mechanism against muscle damage induced by BaCl_2_ (*Figure* *5* *D*). However, *Adck2* ^*+/−*^ myofibres exhibited a higher proportion of myofibres with two, three and four central myonuclei, indicative of an inefficient regeneration capacity and a defective longitudinal alignment of the nuclei within the middle of the myofibres.^40^ Notably, CoQ_10_ administration resulted in a reduction in the number of central nuclei in myofibres (*Figure* *5* *D*).

## Discussion

CoQ deficiency represents a rare pathological condition characterized by a reduction in CoQ levels, classifiable as a mitochondrial disorder. This syndrome exhibits heterogeneity without a clearly defined genotype–phenotype correlation, thereby complicating both diagnosis and prognostic assessment.^17^ The diminution of CoQ can exert adverse effects on multiple organ systems, encompassing the brain (resulting in conditions such as encephalopathy, seizures or cerebellar ataxia), the heart (leading to hypertrophic cardiomyopathy), the kidneys (contributing to nephrotic syndrome) and skeletal muscles (causing myopathy).^16^ Importantly, these organs demonstrate an elevated reliance on mitochondrial OXPHOS. Ageing is a deteriorative and unstoppable functional decline that groups a progressive loss of physiological integrity, an impaired function and an increased vulnerability to disease and death.^S1^ ^,^ ^S2^ Time‐dependent accumulation of cellular damage and loss of biological fitness is considered the main cause of ageing.^S1^ In humans, ADCK2 haploinsufficiency produces an adult‐onset mitochondrial myopathy, defective fatty acid metabolism and premature physical incapacity.^32^ Adult heterozygous *Adck2* mice exhibit a premature metabolic and mitochondrial deregulation^33^ characterized by insulin resistance, impaired mitochondrial respiration in skeletal muscle, lower physical activity and reduced mitochondrial CoQ levels. Alterations found in adult *Adck2* ^*+/−*^ mice resemble the phenotype of old control mice^S3^ ^,^ ^S4^ suggesting premature ageing. Interestingly, we demonstrated here that *Adck2* ^*+/−*^ mice do not display a very pernicious phenotype until adulthood.

We were interested in when the functional decline reported in adult *Adck2* ^*+/−*^ mice starts. Here, we report that a CoQ deficiency model suffers damage during foetal development in mammals, showing the crucial importance of CoQ from the early stages of life. The defects found in mutant embryos are tissue‐specific and are directly connected with the phenotype reported in adult mice.^32^ Previously, a *Caenorhabditis elegans* strain with a deleted coq‐8 gene exhibited arrested embryo development, along with defective tissue development, coinciding with reduced CoQ biosynthesis.^S5^ In particular, certain genes associated with apoptosis or autophagy showed deregulation in the muscle of *Adck2* ^*+/−*^ murine embryos, but prenatal CoQ_10_ administration successfully reversed some of these changes. Together, these results reveal new potential aspects of this syndrome, suggesting the potential existence of defective embryonic development in other CoQ deficiency models.^17^ ^,^ ^S6^

We also hypothesized that CoQ deficiency models with an earlier onset of deleterious changes would suffer more severe damage during development. Oral supplementation of CoQ_10_ is the only treatment that delays the progression of these syndromes, where prompt diagnosis and early initiation of CoQ_10_ administration are crucial.^5^, ^25^ ^,^ ^S7^ Based on the positive effects previously reported from prenatal CoQ_10_ supplementation during pregnancy for embryo development and ovarian fertility^S8^ ^–^ ^S11^ and bearing in mind the defects found specifically in skeletal muscle from embryos, we decided to start a prenatal CoQ_10_ administration. Our observed alterations in the transcriptomic profile suggest a defective structural organization of the muscle and metabolic deregulation. The transcriptomic alterations found in mutant embryos were reverted to a WT profile when CoQ_10_ was administered. Damage in mutant embryos from early development suggests that subsequent CoQ_10_ administration may only partially ameliorate the defects. These results underscore the potential benefits of prenatal CoQ_10_ administration in CoQ deficiency models during embryonic development that could alleviate human diseases associated with genetic defects in CoQ biosynthesis, including nephropathies, ataxias and myopathies.^17^ Chronic and continuous CoQ administration has been previously studied in humans, including in conditions not directly related to defects in CoQ biosynthesis or regulation^S12^ ^,^ ^S13^ or healthy population.^S14^ No negative effects were reported, confirming the safety and tolerance for CoQ. Improvements in plasma markers of oxidative stress and reductions in fatigue were reported, suggesting that chronic CoQ supplementation could be particularly beneficial for these syndromes. Specifically, chronic CoQ administration in CoQ deficiency syndrome has been examined in COQ2, COQ6 and COQ8 patients suffering from nephrotic damage.^S15^ Significant reductions in proteinuria (88% at 12 months) and better preservation of kidney function were found. Finally, a better understanding of the impact of chronic CoQ administration on mitochondria and various tissues should be pursued in murine models to ensure long‐term efficacy and optimal dosing.

Notably, *Adck2* ^*+/−*^ embryos have defective muscle formation, previously associated with a premature onset of robust muscle wasting and sarcopenia.^S16^ We demonstrate a disturbed ageing process in skeletal muscle from *Adck2* ^*+/−*^ mice, affecting metabolic and structural factors. However, manipulations that favour tissue regeneration in postmitotic myofibres may prevent or delay ageing.^S17^ Taking into account the positive preliminary effects of CoQ_10_ administration on skeletal muscle damage^S18^ ^,^ ^S19^ and the decrease in mitochondrial CoQ levels in *Adck2* ^*+/−*^ mice, we administered CoQ_10_ longitudinally to *Adck2* ^*+/−*^ mutant mice. This revealed that CoQ_10_ effectively prevented muscle wasting, fibre‐type changes and grip strength capacity decrease associated with ageing in this genotype. Indeed, old *Adck2* ^*+/−*^ mutant mice have 43% and 32% more grip strength capacity in the two‐ and four‐limb grip tests, respectively, when CoQ_10_ was longitudinally administered, this being a considerable improvement compared to the 28% and 17% that we found when CoQ_10_ was supplemented for a short time.^32^ This improvement cannot be attributed to changes in body mass, as no genotype‐related differences in body mass or tibial length were found during ageing. This represents a novel evaluation of extended and longitudinal CoQ_10_ administration throughout ageing, showcasing its positive impact on skeletal muscle capacity. The current results offer a basis for exploring the effects of CoQ_10_ administration in various skeletal muscle wasting conditions.

Taking into consideration the similarities between prenatal and postnatal myogenesis,^S20^ the recent insights of satellite cell metabolism,^S21^ ^,^ ^S22^ and the demonstration that inner mitochondrial membrane integrity controls satellite cell fate and function,^S23^ ^,^ ^S24^ we assessed satellite cell differentiation in *Adck2* ^*+/−*^ mice. At a young age, a small impairment in satellite cell differentiation was observed in *Adck2* ^*+/−*^ mice because they showed defective mitochondrial function. Metabolic pathways required for skeletal muscle regeneration are dysregulated during ageing.^S25^ Satellite cells from old *Adck2* ^*+/−*^ mice showed a more severe defect during differentiation. Ageing impairs the functions of satellite cells due to inefficient OXPHOS metabolism, altered mitochondrial shape and impaired mitophagy, likely affecting muscle regeneration.^S23^ Our results substantiate this concept by demonstrating a greater deregulation of the OXPHOS system in satellite cells differentiated from old mice. Significantly, this deregulation was ameliorated with longitudinal CoQ_10_ administration. This raises the question of whether CoQ_10_ administration could be beneficial in other satellite cell syndromes and skeletal muscle dystrophies. This is underscored by the demonstration that another OXPHOS substrate, NAD^+^, has the potential to rejuvenate and prevent satellite cell senescence in the mdx (C57BL/10ScSn‐Dmd(mdx)/J) mouse model of muscular dystrophy.^S26^

Non‐functional mitochondria impact skeletal muscle health and function.^S27^ Consistent with this, we show that mitochondria from the skeletal muscle of both young and old *Adck2* ^*+/−*^ mice have defective respiration based on glucose and fatty acid substrates. Despite finding similar mitochondrial mass, mitochondrial function was compromised in skeletal muscle from *Adck2* ^*+/−*^ mice. This difference is likely due to reduced mitochondrial quality, impaired respiratory chain complex activity and metabolic shifts observed in *Adck2* ^*+/−*^, as previously demonstrated in other muscle wasting conditions.^S28^ Defective mitochondria contribute to muscle wasting through deregulation of metabolic pathways.^S29^ We postulated that mitochondria function is defective in *Adck2* ^*+/−*^ from development, consistent with observations in mouse embryonic fibroblasts (MEFs) from this model,^32^ and this defective function could be associated with a CoQ decrease. Notwithstanding, continuous CoQ_10_ supplementation preserved mitochondrial function and safeguarded skeletal muscle from the many deleterious effects of ageing.

Finally, we disrupted skeletal muscle homeostasis by inducing muscle damage in both young and old *Adck2* ^*+/−*^ mice. Upon encountering an injury, satellite cells activate from a quiescent state, initiating a sequence where they undergo proliferation, differentiation and subsequently fusion with damaged myofibres. This process results in the generation of new myonuclei, serving to replace or repair damaged cells.^S30^ ^–^ ^S33^ New nuclei align within the central region of the myofibre during the process of myofibre regeneration.^40^ Remarkably, old *Adck2* ^*+/−*^ skeletal muscle had a higher number of internalised nuclei during regeneration, suggesting an incomplete and defective alignment in the central position of myofibres and a potential delay in regeneration. A higher quantity of internalised nuclei may be linked to the deregulation identified in cytoskeletal pathway expression observed in skeletal muscle from *Adck2* ^*+/−*^ embryos. CoQ_10_ administration reduced the number of internalised nuclei per myofibre, suggesting a normal alignment of the central nuclei. These results suggest a progressive metabolic impairment of skeletal muscle functional capacity in *Adck2* ^*+/−*^ mice, evidencing an age‐related decline. Satellite cells from *Adck2* ^*+/−*^ exhibited decreased metabolic flexibility to efficiently respond to muscle injury. These results position ADCK2 as a putative target for the study of sarcopenia and frailty.

We would like to note that for endpoint experiments, three biological replicates were included, which should be considered a potential limitation of our study. For the isolation of mitochondria from skeletal muscle, a mixture of muscles (including red, white and mixed muscles) was used, as detailed in the Methods section of the supporting information.

In conclusion, our results set forth the idea that CoQ haploinsufficiency models exhibit tissue‐specific defects from development and that these changes become exacerbated during ageing. We anticipate that characterizing these defects in other CoQ deficiency diseases will be critical to harnessing the onset and evolution of reduced CoQ levels. Prenatal and longitudinal CoQ administration may provide new opportunities to treat these disorders and even other myopathies and open further avenues of research in CoQ deficiency.

## Conflict of interest statement

The authors declare not to have any type of conflict of interest.

## Funding

This research was funded by the Instituto de Salud Carlos III (PI20/00541) co‐funded by the European Regional Development Fund ‘A way to make Europe’. This work was supported by the Spanish Ministry of Education, Culture and Sports through Fellowship FPU16/03264 to JDH‐C, an Institutional Grant CEX2020‐001088‐M (María de Maeztu Excellence Unit, Department of Gene Regulation and Morphogenesis at CABD) and PID2020‐117058GB‐I00 from the Spanish Ministry of Science and Innovation (Ministerio de Ciencia e Innovación) to JC, and ProyExcel_00153 from the Andalusian Government Junta de Andalucia (PAIDI 2020; 2021 call) to CV‐G. Relevant funding for the Zammit lab includes the Medical Research Council (MR/P023215/1 and MR/S002472/1), Friends of FSH Research and the FSHD Society (FSHD‐Fall2020‐3308289076). Funding for open access publishing: Universidad Pablo de Olavide/CBUA.

## Supporting information


**Data S1.** Supplementary References.

**Data S2.** Supplementary Methods.

**Table S1.** Primary antibodies.
**Table S2.** Secondary antibodies.
**Table S3.** Primers for qPCR.
**Table S4.** Image analysis pipeline used in Fiji.
**Table S5.** Regression lines for exercise tests.

**Figure S1.** Impact of CoQ_10_ administration in different tissues. A. CoQ_10_ oxidation rate. Reduced and oxidized forms of CoQ_10_ in water were measured by HPLC at different time points during a 9‐day period. B. CoQ_9_, CoQ_10_ levels and ratio CoQ_9_/CoQ_10_ on plasma, spleen and liver from young mice. (*Adck2* ^*+/+*^ N = 3, *Adck2* ^*+/+*^ CoQ_10_ N = 3, *Adck2* ^*+/−*^ N = 3, *Adck2* ^*+/−*^ CoQ_10_ N = 3). C. Representative RNA integrity verification by electrophoresis in an agarose 1% (w/v) gel. D. Representative Stain‐Free gel image used as loading control in Western blotting assays. E. Protein markers of mitochondrial mass and skeletal muscle structural organization in skeletal muscle from 17 d*pc* embryos. (*Adck2* ^*+/+*^ N = 3, *Adck2* ^*+/−*^ N = 3). d*pc*: days *post‐coitum*. Data represent the mean ± SD. One‐way ANOVA test was applied. *p < 0.05; **p < 0.01; ***p < 0.001; ****p < 0.0001.
**Figure S2.** Impact of *Adck2* ablation on the transcriptomic profile of skeletal muscle development. A. Apoptosis pathway genes expression in skeletal muscle from embryos at 17 d*pc* with/without CoQ_10_ prenatal administration. Activated gene expression is represented in green and repressed gene expression, in red (comparisons represented: *Adck2* ^*+/−*^ *vs Adck2* ^*+/+*^, *Adck2* ^*+/−*^ + CoQ_10_ vs *Adck2* ^*+/−*^ and *Adck2* ^*+/−*^ + CoQ_10_ vs *Adck2* ^*+/+*^). N = 3 per group. B. Protein Turnover pathway genes expression in skeletal muscle from embryos at 17 d*pc* on basal conditions and with CoQ_10_ prenatal administration. Activated gene expression is represented in green and repressed gene expression, in red (comparisons represented: *Adck2* ^*+/−*^ *vs Adck2* ^*+/+*^, *Adck2* ^*+/−*^ + CoQ_10_ vs *Adck2* ^*+/−*^ and *Adck2* ^*+/−*^ + CoQ_10_ vs *Adck2* ^*+/+*^). N = 3 per group. C. Mitochondrial biogenesis pathway genes expression in skeletal muscle from embryos at 17 d*pc* with/without CoQ_10_ prenatal administration. Activated gene expression is represented in green and repressed gene expression, in red (comparisons represented: *Adck2* ^*+/−*^ *vs Adck2* ^*+/+*^, *Adck2* ^*+/−*^ + CoQ_10_ vs *Adck2* ^*+/−*^ and *Adck2* ^*+/−*^ + CoQ_10_ vs *Adck2* ^*+/+*^). N = 3 per group. D. Mitochondrial Dynamics and Mitophagy pathways genes expression in skeletal muscle from embryos at 17 d*pc* with/without CoQ_10_ prenatal administration. Activated gene expression is represented in green and repressed gene expression, in red (comparisons represented: *Adck2* ^*+/−*^ *vs Adck2* ^*+/+*^, *Adck2* ^*+/−*^ + CoQ_10_ vs *Adck2* ^*+/−*^ and *Adck2* ^*+/−*^ + CoQ_10_ vs *Adck2* ^*+/+*^). N = 3 per group. E. Volcano Plot representing the transcriptomic profile in skeletal muscle from *Adck2* ^+/−^ + CoQ_10_ vs *Adck2* ^+/+^ +CoQ_10_ of 17 d*pc* embryos. Significant genes (FDR < 0.05) with an altered gene expression higher than 1.75‐fold are coloured in green if activated, or in red if repressed. Gene Ontology functional Enrichment analysis on the genes differentially expressed. N = 3 per group. F. Volcano Plot representing the transcriptomic profile in skeletal muscle from *Adck2* ^+/+^ +CoQ_10_ vs *Adck2* ^+/+^ of 17 d*pc* embryos. Significant genes (FDR < 0.05) with an absolute fold change higher than 1.75‐fold are coloured in green if upregulated, or in red if repressed. Gene Ontology functional Enrichment analysis on the genes differentially expressed. N = 3 per group. G. The gene expression levels, derived from microarray studies (*Adck2* ^+/+^ N = 3, *Adck2* ^+/−^ N = 3, *Adck2* ^+/−^ + CoQ_10_ N = 3) were validated through qPCR (*Adck2* ^+/+^ N = 2, *Adck2* ^+/−^ N = 2, *Adck2*+/− + CoQ_10_  N = 5). d*pc*: days post‐coitum. Data represent the mean ± SD. *p < 0.05; **p < 0.01; ***p < 0.001.
**Figure S3.** Impact of *Adck2* ablation on the development and ageing and on skeletal muscle. A. Representative images of tibialis anterior (TA) muscles from old mice with/without CoQ_10_ administration. TUNEL positive cell immunolabelling (green) and a nuclear DAPI counterstain (blue). Quantification of TUNEL Staining (Ratio: TUNEL + cells/DAPI + cells). (*Adck2* ^*+/+*^ N = 4, *Adck2* ^*+/+*^ +CoQ_10_ N = 4, *Adck2* ^*+/−*^ N = 3, *Adck2* ^*+/−*^ + CoQ_10_ N = 3). Scale bar 50 microns. B. Representative images of young (three‐month‐old) and old (two‐year‐old) mice on standard conditions and under CoQ_10_ supplementation. C. Shinbone length measurements were taken in both young and old mice. The shinbone from the left limb was used for the measurement. Scale bar 500 μm. (Young mice: *Adck2* ^*+/+*^ N = 7, *Adck2* ^*+/+*^ +CoQ_10_ N = 5, *Adck2* ^*+/−*^ N = 5, *Adck2* ^*+/−*^ + CoQ_10_ N = 5). (Old: *Adck2* ^*+/+*^  N= 6, *Adck2* ^*+/+*^ +CoQ_10_ N = 5, *Adck2* ^*+/−*^ N= 8, *Adck2* ^*+/−*^ + CoQ_10_ N = 9). D. Myofibers cross‐sectional area (left) and minimal Feret's diameter (right) distribution in young mice with/without CoQ_10_ administration. The x‐axis represents the area or the diameter, and the y‐axis represents the percentage of the myofibers with specific dimensions. (*Adck2* ^*+/+*^ N = 4, *Adck2* ^*+/+*^ +CoQ_10_ N = 4, *Adck2* ^*+/−*^ N = 4, *Adck2* ^*+/−*^ + CoQ_10_ N = 4).E. Myofibers cross‐sectional area (left) and minimal Feret's diameter (right) distribution in old mice with/without CoQ_10_ administration. The x‐axis represents the area or the diameter, and the y‐axis represents the percentage of the myofibers with specific dimensions. (*Adck2* ^*+/+*^ N = 4, *Adck2* ^*+/+*^ +CoQ_10_ N = 4, *Adck2* ^*+/−*^ N = 4, *Adck2* ^*+/−*^ + CoQ_10_ N = 3).
**Figure S4.** Impact of *Adck2* in skeletal muscle in young and old mice. A. Representative whole transversal muscle section used for the analysis of myofiber size in young mice with/without CoQ_10_ administration, immunolabelled for dystrophin to delimit the myofibres (green) with a DAPI nuclear counterstain (blue). B. Representative whole transversal muscle section used for the analysis of myofiber size in old mice with/without CoQ_10_ administration, immunolabelled for dystrophin to delimit the myofibres (green) with a DAPI nuclear counterstain (blue). C. Representative whole transversal muscle section used for the analysis of myofiber type in young mice with/without CoQ_10_ administration, immunolabelled for Type IIa (green), IIb (red), and IIx myofibers (unlabelled). D. Representative whole transversal muscle section used for the analysis of myofiber type in old mice with/without CoQ_10_ administration, immunolabelled for Type IIa (green), IIb (red), and IIx myofibers (unlabelled). Scale bar 500 microns.
**Figure S5.** Skeletal muscle structure and function of heterozygous *Adck2* during ageing. A. Minimal Feret's diameter quantification for the complete transversal section of TA muscle in young mice with/without CoQ_10_ administration. Distribution of myofibers in function of the size. (*Adck2* ^*+/+*^ N = 4, *Adck2* ^*+/+*^ +CoQ_10_ N = 4, *Adck2* ^*+/−*^ N = 4, *Adck2* ^*+/−*^ + CoQ_10_ N = 4). B. Minimum Feret's diameter quantification for the complete transversal section of TA muscle in old mice with/without CoQ_10_ administration. Distribution of myofibers in function of the size. (*Adck2* ^*+/+*^ N = 4, *Adck2* ^*+/+*^ +CoQ_10_ N = 4, *Adck2* ^*+/−*^ N = 4, *Adck2* ^*+/−*^ + CoQ_10_ N = 3). C. Results for four‐limb grip strength tests through ageing with and without body weight normalization. (Young mice: *Adck2* ^*+/+*^ N = 8, *Adck2* ^*+/+*^ +CoQ_10_ N = 3, *Adck2* ^*+/−*^ N = 11, *Adck2* ^*+/−*^ + CoQ_10_ N = 3). (Adult mice: *Adck2* ^*+/+*^ N = 16, *Adck2* ^*+/+*^ +CoQ_10_ N = 5, *Adck2* ^*+/−*^ N = 9, *Adck2* ^*+/−*^ + CoQ_10_ N = 15). (Old adult mice: *Adck2* ^*+/+*^ N = 8, *Adck2* ^*+/+*^ +CoQ_10_ N = 10, *Adck2* ^*+/−*^ N = 15, *Adck2* ^*+/−*^ + CoQ_10_ N = 9). (Old: *Adck2* ^*+/+*^ N = 7, *Adck2* ^*+/+*^ +CoQ_10_ N = 5, *Adck2* ^*+/−*^ N = 20, *Adck2* ^*+/−*^ + CoQ_10_ N = 9). D. Results for weight lifting tests through ageing with and without body weight normalization. (Young mice: *Adck2* ^*+/+*^ N = 8, *Adck2* ^*+/+*^ +CoQ_10_ N = 2, *Adck2* ^*+/−*^ N = 8, *Adck2* ^*+/−*^ + CoQ_10_ N = 3). (Adult mice: *Adck2* ^*+/+*^ N = 22, *Adck2* ^*+/+*^ +CoQ_10_ N = 11, *Adck2* ^*+/−*^ N = 22, *Adck2* ^*+/−*^ + CoQ_10_ N = 23). (Old adult mice: *Adck2* ^*+/+*^ N = 23, *Adck2* ^*+/+*^ +CoQ_10_ N = 14, *Adck2* ^*+/−*^ N = 20, *Adck2* ^*+/−*^ + CoQ_10_ N = 18). (Old: *Adck2* ^*+/+*^ N = 7, *Adck2* ^*+/+*^ +CoQ_10_ N = 7, *Adck2* ^*+/−*^ N = 14, *Adck2* ^*+/−*^ + CoQ_10_ N = 6). E. Results for two‐limb grip strength tests without body weight normalization. (Young mice: *Adck2* ^*+/+*^ N = 8, *Adck2* ^*+/+*^ +CoQ_10_ N = 3, *Adck2* ^*+/−*^ N = 11, *Adck2* ^*+/−*^ + CoQ_10_ N = 3). (Adult mice: *Adck2* ^*+/+*^ N = 16, *Adck2* ^*+/+*^ +CoQ_10_ N = 6, *Adck2* ^*+/−*^ N = 9, *Adck2* ^*+/−*^ + CoQ_10_ N = 15). (Old adult mice: *Adck2* ^*+/+*^ N = 8, *Adck2* ^*+/+*^ +CoQ_10_ N = 10, *Adck2* ^*+/−*^ N = 15, *Adck2* ^*+/−*^ + CoQ_10_ N = 9). (Old: *Adck2* ^*+/+*^ N = 7, *Adck2* ^*+/+*^ +CoQ_10_ N = 5, *Adck2* ^*+/−*^ N = 23, *Adck2* ^*+/−*^ + CoQ_10_ N = 10). F. Body weight through ageing. (Young mice: *Adck2* ^*+/+*^ N = 10, *Adck2* ^*+/+*^ +CoQ_10_ N = 3, *Adck2* ^*+/−*^ N = 10, *Adck2* ^*+/−*^ + CoQ_10_ N = 3). (Adult mice: *Adck2* ^*+/+*^ N = 15, *Adck2* ^*+/+*^ +CoQ_10_ N = 5, *Adck2* ^*+/−*^ N = 9, *Adck2* ^*+/−*^ + CoQ_10_ N = 15). (Old adult mice: *Adck2* ^*+/+*^ N = 9, *Adck2* ^*+/+*^ +CoQ_10_ N = 10, *Adck2* ^*+/−*^ N = 15, *Adck2* ^*+/−*^ + CoQ_10_ N = 8). (Old: *Adck2* ^*+/+*^ N = 7, *Adck2* ^*+/+*^ +CoQ_10_ N = 5, *Adck2* ^*+/−*^ N = 15, *Adck2* ^*+/−*^ + CoQ_10_ N = 10). Data represent the mean ± SD. One‐way ANOVA test was applied. *p < 0.05; **p < 0.01; ***p < 0.001; ****p < 0.0001.
**Figure S6.** Voluntary wheel running activity of heterozygous *Adck2* during ageing. A. Voluntary wheel activity in the automated home cage phenotyping system in young mice with/without CoQ_10_ administration. (*Adck2* ^*+/+*^ N = 6, *Adck2* ^*+/+*^ +CoQ_10_ N = 6, *Adck2* ^*+/−*^ N = 6, *Adck2* ^*+/−*^ + CoQ_10_ N = 6). B. Voluntary wheel activity in the automated home cage phenotyping system in adult mice with/without CoQ_10_ administration. (*Adck2* ^*+/+*^ N = 8, *Adck2* ^*+/+*^ +CoQ_10_ N = 6, *Adck2* ^*+/−*^ N = 7, *Adck2* ^*+/−*^ + CoQ_10_ N = 5). C. Voluntary wheel activity in the automated home cage phenotyping system in old adult mice with/without CoQ_10_ administration. (*Adck2* ^*+/+*^ N = 6, *Adck2* ^*+/+*^ +CoQ_10_ N = 4, *Adck2* ^*+/−*^ N = 6, *Adck2* ^*+/−*^ + CoQ_10_ N = 5). D. Voluntary wheel activity in the automated home cage phenotyping system in old mice with/without CoQ_10_ administration. (*Adck2* ^*+/+*^ N = 4, *Adck2* ^*+/+*^ +CoQ_10_ N = 5, *Adck2* ^*+/−*^ N = 5, *Adck2* ^*+/−*^ + CoQ_10_ N = 4). Data are expressed as distance recorded in centimetres (cm). In all cases, data represent the mean ± SD. One‐way ANOVA test was applied. *p < 0.05; **p < 0.01.
**Figure S7.** Impact of *Adck2* ablation on mitochondrial content, dynamics and mitophagy. A. Protein markers of mitochondrial mass in skeletal muscle from young mice. (Young mice: *Adck2* ^*+/+*^ N = 3, *Adck2* ^*+/+*^ +CoQ_10_ N = 3, *Adck2* ^*+/−*^ N = 3, *Adck2* ^*+/−*^ + CoQ_10_ N = 3). B. Protein markers of mitochondrial dynamics and mitophagy in skeletal muscle from young mice. (Young mice: *Adck2* ^*+/+*^ N = 3, *Adck2* ^*+/+*^ +CoQ_10_ N = 3, *Adck2* ^*+/−*^ N = 3, *Adck2* ^*+/−*^ + CoQ_10_ N = 3). C. Representative images of myogenic cells used to evaluate mitochondrial morphology. Scale bar 20 microns. D. Parameters represented area, perimeter, minimum Feret diameter and circularity. (*Adck2* ^*+/+*^ N = 12, *Adck2* ^*+/+*^ +CoQ_10_  N = 9, *Adck2* ^*+/−*^ N = 18, *Adck2* ^*+/−*^ + CoQ_10_ N = 26). Data represent the mean ± SD. One‐way ANOVA test was applied. *p < 0.05; **p < 0.01; ***p < 0.001; ****p < 0.0001.
**Figure S8.** Mitochondrial bioenergetic assessment through ageing. A. 1D BN‐PAGE for CI in skeletal muscle mitochondria isolated from old mice with/without CoQ_10_ administration. B. 1D BN‐PAGE for CII in skeletal muscle mitochondria isolated from old mice with/without CoQ_10_ administration. C. 1D BN‐PAGE for CIII in skeletal muscle mitochondria isolated from old mice with/without CoQ_10_ administration. D. 1D BN‐PAGE for CIV in skeletal muscle mitochondria isolated from old mice with/without CoQ_10_ administration. E. 1D BN‐PAGE for CV in skeletal muscle mitochondria isolated from old mice with/without CoQ_10_ administration. F. CoQ_9_/CoQ_10_ ratio on skeletal muscle mitochondria from young and old mice Data represent the mean ± SD (Young mice: *Adck2* ^*+/+*^ N = 6, *Adck2* ^*+/+*^ CoQ_10_ N = 4, *Adck2* ^*+/−*^ N = 5, *Adck2* ^*+/−*^ CoQ_10_ N = 5). (Old mice: *Adck2* ^*+/+*^ N = 11, *Adck2* ^*+/+*^ CoQ_10_ = 10, *Adck2* ^*+/−*^ N = 9, *Adck2* ^*+/−*^ CoQ_10_ N = 7). G. *Adck2* expression levels in skeletal muscle from young and old mice. (Young mice: *Adck2* ^*+/+*^ N = 3, *Adck2* ^*+/+*^ CoQ_10_ N = 3, *Adck2* ^*+/−*^ N = 3, *Adck2* ^*+/−*^ CoQ_10_ N = 3). (Old mice: *Adck2* ^*+/+*^ N = 3, *Adck2* ^*+/+*^ CoQ_10_ N = 4, *Adck2* ^*+/−*^ N = 4, *Adck2* ^*+/−*^ CoQ_10_ N = 3). 1D BN‐PAGE (Mono‐dimensional blue native polyacrylamide gel electrophoresis). Data represent the mean ± SD. One‐way ANOVA test was applied. *p < 0.05; **p < 0.01.
**Figure S9.** The effect of *Adck2* on skeletal muscle regeneration in young and old mice. A. Representative whole transversal muscle section used for the analysis of skeletal muscle regeneration 14 dpi in young mice with/without CoQ_10_ administration, immunolabelled for dystrophin to delimit the myofibres (green) with a DAPI nuclear counterstain (blue). The yellow box represents the section included in the main figure. B. Representative whole transversal muscle section used for the analysis of skeletal muscle regeneration 14 dpi in young mice with/without CoQ_10_ administration, immunolabelled for dystrophin to delimit the myofibres (green) with a DAPI nuclear counterstain (blue). Scale bar 500 microns. The yellow box represents the section included in the main figure.
