# Supplementary material for: Prenatal and progressive coenzyme Q10 administration to mitigate muscle dysfunction in mitochondrial disease
Source: J Cachexia Sarcopenia Muscle. 2024 Oct 2;15(6):2402–16. doi: 10.1002/jcsm.13574 (PMC11634497; doi:10.1002/jcsm.13574)
Supplement: Supplementary file 1 — Data S1. Supplementary References. [file JCSM-15-2402-s002.docx]

**Supplemental References**

**Title:** Prenatal and progressive Coenzyme Q_10_ administration to mitigate muscle dysfunction in mitochondrial disease.

Juan Diego Hernández-Camacho^1,2*^, Cristina Vicente-García^1^, Lorena Ardila-García^1^, Ana Padilla-Campos^1^, Guillermo López-Lluch^1,2^, Carlos Santos-Ocaña^1,2^, Peter S. Zammit^3^, Jaime J. Carvajal^1^, Plácido Navas^1,2^ and Daniel J. M. Fernández-Ayala^1,2*^.

1 Centro Andaluz de Biología del Desarrollo-CSIC, Universidad Pablo de Olavide, ES-41013 Seville, Spain

2 CIBERER, Instituto de Salud Carlos III, Madrid, Spain.

3 King's College London, Randall Centre for Cell and Molecular Biophysics, London, SE1 1UL, UK.

*Correspondence to: jdhercam@alu.upo.es and dmorfer@upo.es

Supplemental References

S1. López-Otín C, Blasco MA, Partridge L, Serrano M, Kroemer G. The hallmarks of aging. Cell. 2013;153(6):1194-217.

S2. López-Otín C, Blasco MA, Partridge L, Serrano M, Kroemer G. Hallmarks of aging: An expanding universe. Cell. 2023;186(2):243-278.

S3. Distefano G, Goodpaster BH. Effects of exercise and aging on skeletal muscle. Cold Spring Harb Perspect Med. 2018;8(3)

S4. Miwa S, Kashyap S, Chini E, von Zglinicki T. Mitochondrial dysfunction in cell senescence and aging. J Clin Invest. 2022;132(1)

S5. Asencio C, Navas P, Cabello J, Schnabel R, Cypser JR, et al. Coenzyme Q supports distinct developmental processes in Caenorhabditis elegans. Mech Ageing Dev. 2009;130(3):145-153.

S6. Alcázar-Fabra M, Rodríguez-Sánchez F, Trevisson E, Brea-Calvo G. Primary Coenzyme Q deficiencies: A literature review and online platform of clinical features to uncover genotype-phenotype correlations. Free Radic Biol Med. 2021;167:141-180.

S7. Doimo M, Desbats MA, Cerqua C, Cassina M, Trevisson E, et al. Genetics of coenzyme q10 deficiency. Mol Syndromol. 2014;5(3-4):156-162.

S8. Xu Y, Nisenblat V, Lu C, Li R, Qiao J, et al. Pretreatment with coenzyme Q10 improves ovarian response and embryo quality in low-prognosis young women with decreased ovarian reserve: a randomized controlled trial. Reprod Biol Endocrinol. 2018;16(1):29.

S9. Liang S, Niu YJ, Shin KT, Cui XS. Protective effects of coenzyme Q10 on developmental competence of porcine early embryos. Microsc Microanal. 2017;23(4):849-858.

S10. Giorgio V, Schiavone M, Galber C, Carini M, Da Ros T, et al. The idebenone metabolite QS10 restores electron transfer in complex I and coenzyme Q defects. Biochim Biophys Acta Bioenerg. 2018;1859(10):901-908.

S11. Ben-Meir A, Burstein E, Borrego-Alvarez A, Chong J, Wong E, et al. Coenzyme Q10 restores oocyte mitochondrial function and fertility during reproductive aging. Aging Cell. 2015;14(5):887-895.

S12. Rivara MB, Yeung CK, Robinson-Cohen C, Phillips CR, Ruzinski J, et al. Effect of coenzyme Q10 on biomarkers of oxidative stress and cardiac function in hemodialysis patients: The CoQ10 biomarker trial. Am J Kidney Dis. 2017;69(3):389-399.

S13. Castro-Marrero J, Segundo MJ, Lacasa M, Martinez-Martinez A, Sentañes RS, et al. Effect of dietary coenzyme Q10 plus NADH supplementation on fatigue perception and health-related quality of life in individuals with myalgic encephalomyelitis/chronic fatigue syndrome: A prospective, randomized, double-blind, placebo-controlled trial. Nutrients. 2021;13(8):2658.

S14. Cicero AFG, Fogacci F, Di Micoli A, Veronesi M, Borghi C. Noninvasive instrumental evaluation of coenzyme Q10 phytosome on endothelial reactivity in healthy nonsmoking young volunteers: A double‐blind, randomized, placebo‐controlled crossover clinical trial. Biofactors. 2022;48(6):1160-1165.

S15. Drovandi S, Lipska-Ziętkiewicz BS, Ozaltin F, Emma F, Gulhan B, et al. Oral Coenzyme Q10 supplementation leads to better preservation of kidney function in steroid-resistant nephrotic syndrome due to primary Coenzyme Q10 deficiency. Kidney Int. 2022;102(3):604-612.

S16. McKenna CF, Fry CS. Altered satellite cell dynamics accompany skeletal muscle atrophy during chronic illness, disuse, and aging. Curr Opin Clin Nutr Metab Care. 2017;20(6):447-452.

S17. García-Prat L, Sousa-Victor P, Muñoz-Cánoves P. Functional dysregulation of stem cells during aging: a focus on skeletal muscle stem cells. FEBS J. 2013;280(17):4051-4062.

S18. Ovchinnikov AN, Paoli A, Seleznev VV, Deryugina AV. Royal jelly plus coenzyme Q10 supplementation improves high-intensity interval exercise performance via changes in plasmatic and salivary biomarkers of oxidative stress and muscle damage in swimmers: a randomized, double-blind, placebo-controlled pilot trial. J Int Soc Sports Nutr. 2022;19(1):239-257.

S19. Drobnic F, Lizarraga MA, Caballero-García A, Cordova A. Coenzyme Q10 supplementation and its impact on exercise and sport performance in humans: A recovery or a performance-enhancing molecule? Nutrients. 2022;14(9):1811.

S20. Morgan J, Partridge T. Skeletal muscle in health and disease. Dis Model Mech. 2020;13(8)

S21. Relaix F, Bencze M, Borok MJ, Der Vartanian A, Gattazzo F, et al. Perspectives on skeletal muscle stem cells. Nat Commun. 2021;12(1):692.

S22. Kimoloi S, Sen A, Guenther S, Braun T, Brügmann T, et al. Combined fibre atrophy and decreased muscle regeneration capacity driven by mitochondrial DNA alterations underlie the development of sarcopenia. J Cachexia Sarcopenia Muscle. 2022;13(4):2132-2145.

S23. Hong X, Isern J, Campanario S, Perdiguero E, Ramírez-Pardo I, et al. Mitochondrial dynamics maintain muscle stem cell regenerative competence throughout adult life by regulating metabolism and mitophagy. Cell Stem Cell. 2022;29(10):1506-1508.

S24. Baker N, Wade S, Triolo M, Girgis J, Chwastek D, et al. The mitochondrial protein OPA1 regulates the quiescent state of adult muscle stem cells. Cell Stem Cell. 2022;29(8):1315-1332.e9.

S25. Luo L, Chua YB, Liu T, Liang K, Chua MJ, et al. Muscle injuries induce a prostacyclin-PPARγ/PGC1a-FAO spike that boosts regeneration. Adv Sci (Weinh). 2023;10(10)

S26. Zhang H, Ryu D, Wu Y, Gariani K, Wang X, et al. NAD+ repletion improves mitochondrial and stem cell function and enhances life span in mice. Science. 2016;352(6292):1436-1443.

S27. Chen TH, Koh KY, Lin KM, Chou CK. Mitochondrial dysfunction as an underlying cause of skeletal muscle disorders. Int J Mol Sci. 2022;23(21):12926.

S28. Acin-Perez R, Benincá C, Fernandez Del Rio L, Shu C, Baghdasarian S, et al. Inhibition of ATP synthase reverse activity restores energy homeostasis in mitochondrial pathologies. EMBO J. 2023;42(6)

S29. Dark C, Ali N, Golenkina S, Dhyani V, Blazev R, et al. Mitochondrial fusion and altered beta-oxidation drive muscle wasting in a Drosophila cachexia model. EMBO Rep. 2024;25(2):1835-1858.

S30. Relaix F, Zammit PS. Satellite cells are essential for skeletal muscle regeneration: the cell on the edge returns centre stage. Development. 2012;139(16):2845-2856.

S31. Schaaf GJ, van Gestel TJM, In 't Groen SLM, de Jong B, Boomaars B, et al. Satellite cells maintain regenerative capacity but fail to repair disease-associated muscle damage in mice with Pompe disease. Acta Neuropathol Commun. 2018;6(1):119.

S32. Hauck JS, Howard ZM, Lowe J, Rastogi N, Pico MG, et al. Mineralocorticoid receptor signaling contributes to normal muscle repair after acute injury. Front Physiol. 2019;10:1324.

S33. Chaweewannakorn C, Tsuchiya M, Koide M, Hatakeyama H, Tanaka Y, et al. Roles of IL-1α/β in regeneration of cardiotoxin-injured muscle and satellite cell function. Am J Physiol Regul Integr Comp Physiol. 2018;315(1)

S34. von Haehling S, Coats AJS, Anker SD. Ethical guidelines for publishing in the Journal of Cachexia, Sarcopenia and Muscle: update 2021. J Cachexia Sarcopenia Muscle. 2021;12(5):2259-2261.
