# Supplementary material for: Prenatal and progressive coenzyme Q10 administration to mitigate muscle dysfunction in mitochondrial disease
Source: J Cachexia Sarcopenia Muscle. 2024 Oct 2;15(6):2402–16. doi: 10.1002/jcsm.13574 (PMC11634497; doi:10.1002/jcsm.13574)
Supplement: Supplementary file 2 — Data S2. Supplementary Methods. [file JCSM-15-2402-s003.docx]

**Supplementary Methods**

**Title:** Prenatal and progressive Coenzyme Q_10_ administration to mitigate muscle dysfunction in mitochondrial disease.

Juan Diego Hernández-Camacho^1,2*^, Cristina Vicente-García^1^, Lorena Ardila-García^1^, Ana Padilla-Campos^1^, Guillermo López-Lluch^1,2^, Carlos Santos-Ocaña^1,2^, Peter S. Zammit^3^, Jaime J. Carvajal^1^, Plácido Navas^1,2^ and Daniel J. M. Fernández-Ayala^1,2*^.

1 Centro Andaluz de Biología del Desarrollo-CSIC, Universidad Pablo de Olavide, ES-41013 Seville, Spain

2 CIBERER, Instituto de Salud Carlos III, Madrid, Spain.

3 King's College London, Randall Centre for Cell and Molecular Biophysics, London, SE1 1UL, UK.

*Correspondence to: jdhercam@alu.upo.es and dmorfer@upo.es

**Mouse Model, Study Approval, Mouse Breeding and CoQ_10_ administration**

Mouse housing and sample collection were conducted in accordance with the guidelines outlined in Spanish Royal Decree 53/2013, European Directive 2010/63/EU, and other relevant guidelines. As well as protocols approved by the Universidad Pablo de Olavide Ethics Committee, Seville, Spain (authorization no 12/03/2021/033).

The *Adck2* knockout mouse model was generated in the C57BL/6J background, as previously described (1). Mice were bred and raised in the animal facility at the Centro Andaluz de Biología del Desarrollo (CABD) and housed with male littermates in autoclavable polycarbonate cages, with *ad libitum* access to food and autoclaved water. The environmental conditions of housing were 12 h light/dark cycle. The light period started at 08:00 a.m. Temperature and humidity oscillated within the range of 22ºC ± 2ºC and 54 ± 1%, respectively. The diet of mice was SAFE D40 (Scientific Animal Food and Engineering). Mice were examined at various ageing stages: young mice (3 month-old), adult mice (12 month-old), old adult mice (18 month-old) and old mice (24 month-old). Mice were sacrificed by cervical dislocation. To assess body development and aging, tibia length was measured in three-month-old and two-year-old mice using scopes with a DDC digital camera. The left lower limb was removed from each animal and immersed in 2% w/v NaOH for 7-9 days to remove the skin, skeletal muscle, and fat from the bones.

Reduced Coenzyme Q_10_ (CoQ_10_) (Kaneka QH stabilized powder type P30) (Kaneka Pharma Europe, Brussels, Belgium) was formulated in nanoparticles and dissolved in the water bottles. The mean water consumption from mice was previously calculated (2-3 ml/day). CoQ_10_ was dissolved in water (0.5 mg/ml) to achieve a final mean ingestion of 33.33-50 mg/kg/day per mouse, in accordance with the doses normally used in CoQ deficient patients(2–4). The CoQ_10_ oxidation rate in water was calculated by HPLC (High-Performance Liquid Chromatography) (*Figure S1A*) showing an increase in oxidation after 2 days. Therefore, to avoid oxidation and ensure that most of the CoQ_10_ ingested by mice was in its reduced form, fresh water with CoQ_10_ dissolved was provided to mice three times per week. Non-supplemented age-matched mice under the same environmental conditions were used as control.

CoQ_10_ administration was initiated in adult mice and continued in their progeny for two generations to guarantee that mice were developed under CoQ_10_ administration. After CoQ_10_ administration for two generations, mice were longitudinally supplemented throughout their entire life to assess the effect of CoQ_10_ on development, postnatal growth and ageing. After CoQ_10_ supplementation for two previous generations, CoQ levels were assessed in WT (wild type) and *Adck2^+/-^* 3-month-old mice finding a significant increase in plasma and different tissues and a change in the CoQ_9_/CoQ_10_ ratio (*Figure S1B*).

**Physical Performance Assessment**

All tests were conducted at the same time of the day (2:00 p.m.) to reduce variability related to circadian rhythms. To minimize intra-evaluator variability, the same examiner always performed mouse manipulation. All mice were brought to the experimental room for acclimatization 30 min before starting any tests.

The grip strength test was used to examine *in vivo* muscle force with the forelimbs or with all limbs (Grip Strength Columbus apparatus, Columbus Instruments, OH, United States) (5). Mice were held by the middle of the tail and lowered over the grid, to allow them to grasp it tightly. Each mouse was evaluated five times, and the three more similar results were used for analysis.

For the weight-lifting test (6), mice were held by the middle of the tail and allowed to grasp steel mesh balls attached to increasingly heavier weights, scoring a value depending on the weight (16 g, value = 1; 32 g, value = 2; 48 g, value = 3; 64 g, value = 4; and 80 g, value = 5). The test was considered passed, if the mouse held the weight for 3 s and a new, heavier weight was offered, whereas if the mouse dropped the weight before 3 s, the total time (in seconds) was noted. Mice were allowed 3 attempts to pass the test with each weight, giving each weight the greatest number of seconds without dropping it. The final score was calculated as the product of the value for the heaviest weight held for more than 1 s, multiplied by the time (seconds), and normalized to body weight.

**Automated Phenotyping**

An automated home cage phenotyping system (PhenoMaster TSE system, TSE Systems GmbH, Hessen; Germany) was used to further characterize the mouse phenotype under controlled conditions (7). The phenotyping system allows to record *in vivo* parameters during both day and night periods. Mice from all groups studied were included in every experiment. The first 36 h of each experiment were considered as acclimatization period and were excluded from analysis. Data from 3 day/night cycles from the different mice were collected for analysis.

**Microarray Analysis**

Transcriptome analyses were conducted in triplicate in foetal liver, brain and skeletal muscles (extracted from the four limbs of the embryo) at 17 days of development. Specifically, we decided to focus on 17 days to explore a developmental stage where the studied organs are fully functional and close to the end of gestation (average length in mice is 19.5 days). For skeletal muscle, secondary fibers are already formed at this developmental stage which are essential for the growth and functional maturation of skeletal muscle (8,9). At this stage of development, the liver has progressed significantly in its development and structural maturation, forming various cell types including portal mesenchyme cells, endothelial cells, hepatoblasts, and biliary epithelium (10,11). The temporal and spatial gene expression of mouse brain development has demonstrated that important processes such as neuronal diversity and maturation has occurred at this developmental stage (12). Results from heterozygous *Adck2^+/-^* embryos were compared to *Adck2^+/+^* independently for each tissue. For sample collection, pregnant females were sacrificed in the morning (09:00 am), and embryos were carefully collected under a dissection microscope and placed on cold phosphate-buffered saline (PBS). Embryos were meticulously separated from the umbilical cord and the placenta. The brain, the liver and the muscles from the four limbs were carefully dissected. Homogenization of the tissues was performed in 1 ml Trizol (Invitrogen) using a FastPrep-24 5G homogenizer (MP Biolabs) in Lysing Matrix D tubes of 1.4 mm ceramic spheres. Then, samples were incubated on ice for 5 min to allow complete dissociation of the nucleoprotein complexes. Next, 0.2 ml of chloroform (PamRear/AppliChem) was added per tube, mixed by agitation and incubated for 2-3 min at room temperature (RT). Samples were centrifugated at 12000 g for 15 min and the aqueous colourless upper phase was carefully collected. Isopropanol (PamRear/AppliChem) (0.5 ml) was added, mixed and incubated for 10 min at RT to precipitate RNA. Samples were centrifugated at 12000g for 10 min at 4ºC and the supernatant was discarded. The pellet was washed with ethanol (PamRear/AppliChem) 75% (v/v) and left to dry. The RNA pellet was solubilized in 30 μl RNase-free water and stored at -80ºC.

RNA concentration and purity were determined spectrophotometrically in a Nanodrop (2000, ThermoFisher) and RNA integrity was verified by electrophoresis in an agarose 1% (w/v) gel (*Figure S1C*). RNA was cleaned using the RNeasy MinElute Cleanup Kit (Quiagen) according to the manufacturer's instructions and finally, RNA concentration was quantified in a Bioanalyzer (Agilent). Samples were further processed at the Genomic Unit of CABIMER (C/Américo Vespucio 24, 41092 Seville, Spain). A cRNA probe was synthesized and fragmented from each RNA sample using Affymetrix/Thermofisher protocols and kits provided by QIAGEN. Each cRNA probe was then hybridized into independent Mouse Clarion D arrays. Hybridization and scanning of the arrays were performed according to manufacturer’s procedures. Computational analysis was performed using the Transcriptome Analysis Console (TAC) Software (Affymetrix/ThermoFisher). Data were prefiltered according to their absolute fold-change (higher than 1.75, at linear scale) and their corrected FDR p-value (lower than 0.05) (13). Deregulated genes were selected and classified according to their Gene Ontology (GO) by WebGestalt software (<https://www.webgestalt.org/>). Transcriptomic data have been deposited at National Center for Biotechnology Information, Gene Expression Omnibus database repository (NCBI-GEO) with the dataset identifier GSE253654.

**Analysis of CoQ levels**

Mitochondria were isolated from skeletal muscle through serial centrifugations under osmotic conditions as previously described (14). Pellets of 100 μg of mitochondria-enriched proteins were resuspended in 170 μl of PBS and 10 μl of 10 μM CoQ_6_ was included as an internal loss control. Sodium dodecyl sulphate (SDS) (PamRear/AppliChem) was added to a final concentration of 1% (w/v) to rupture cellular membranes and the mixture vortexed for 1 min. Then, 300 μl of ethanol-isopropanol (90:10 v/v) were added and vortexed for 1 min. Hexane (PamRear/AppliChem) (700 μl) was added, vortexed for 1 min, and followed by centrifugation at 1000 g for 5 min at 4ºC. The upper phase, containing the lipids, was transferred to a new Eppendorf tube, and the hexane extraction step was repeated twice. Finally, hexane was evaporated using a Speed Vac Vacuum concentrator (Savant#SPD121P) and the lipid extract was injected into a HPLC system to determine CoQ content as previously described(15).

**Western Blotting and Immunodetection**

Skeletal muscle (500 mg) or hindlimb skeletal muscle in case of embryos was homogenized by sonication in RIPA buffer [Tris-HCL 50 mM pH=7.4, NaCl 150 mM, EDTA 1 mM, 1% (v/v) NP40, Na_3_VO_4_ 5 mM, Na_4_P_2_O_7_ 10 mM, protease inhibitor cocktail (PIC) 1X]. The lysate was then centrifuged to discard unhomogenized tissue, and the supernatant was transferred to a new tube. After protein quantification, 40 μg or 20 μg of proteins were mixed with loading buffer (LB) 1X [10% (w/v) SDS, dithiothreitol (DTT) 500 mM, 50% (v/v) Glycerol, Tris-HCL 250 mM and 0.5% (w/v) bromophenol blue dye, pH=6.8] and with DTT (200 mM), loaded in a polyacrylamide gel (TGX Stain-Free FastCast Acrylamide Kit, 7.5%, 10% and 12%. Bio-Rad, Hercules, CA, United States) and run in electrophoresis under denaturing conditions (SDS-PAGE), for 40 min at 200 V using a Mini-PROTEAN Tetra cell (Bio-Rad, Hercules, CA, United States) in 1X Tris/Glycine/SDS (TGS) buffer. The gel was then activated by ultraviolet (UV) exposure using a Bio-Rad Chemidoc imager, and the Stain-Free gel image was used as loading control (16) (*Figure S1D*). The gel was transferred onto a 0.2 μm nitrocellulose membrane, using a Trans-Blot Turbo Transfer (Bio-Rad). The membrane was blocked with 5% milk powder (blotting-Grade Blocker; BioRad Hercules, CA, United States) in Tween-Tris-buffered saline (TTBS) [20 mM (w/v) Tris-HCL, 0.15 M (w/v) NaCl, 0.5% (v/v) Tween20, pH=7.4] for 1 h at RT, and incubated overnight (OV) with specific primary antibodies (Supplementary Table 1) diluted in blocking solution. Next, the membrane was washed three times in TTBS and incubated for 2 h at RT with the appropriate secondary antibodies (Supplementary Table 2) conjugated with horseradish peroxidase (HRP). Finally, the membrane was incubated with Crescendo Western HRP substrate (Milipore Burlington, MA, United States) and exposed using a ChemiDoc TM XRS + Imaging System (BioRad, Hercules, CA, United States). Band Intensity was determined with Image Lab Software (BioRad, Hercules, CA, United States).

**1D BN-PAGE for skeletal muscle mitochondria**

1D BN-PAGE was conducted as previously described (17) with some modifications. Briefly, mitochondria were purified from skeletal muscle through serial centrifugations under osmotic conditions as described (14). Digitonin (200 μg) was homogenized in BT+AC buffer (Bis-Tris [75mM] pH=7.4 and aminocaproic acid 1.5 M) and heated to 95ºC for 2 min. Next, 50 μg of protein pellets of enriched skeletal muscle mitochondria were resuspended in 30 µl of digitonin solution (ratio 4:1) and incubated on ice for 10 minutes. The mixture was then centrifugated at 16000 g for 20 min and the resulting supernatant was transferred to a new tube. Loading buffer 4x (Coomassie Blue 5%) (Invitrogen) was added to 25 μL supernatant. The digitonin solubilized mitochondria were loaded onto a 3-12% Bis-Tris Invitrogen^TM^ Novex^TM^ NativePAGE^TM^ acrylamide gel (1mm) (Invitrogen, BN2011BX10). The samples migrated using anode running buffer (Invitrogen, BN2001) supplemented with Cathode Buffer Additive (0.5%) (Invitrogen, BN2002) at 200V for 4 hours. Subsequently, the gels were transferred onto polyvinylidene difluoride (PVDF) membranes in transfer buffer (NaHCO_3_ [10 mM] and NaCO_3_ [3 mM]) at 300mA 20V for 2 h. After transfer, membranes were washed with methanol to remove Coomassie blue staining. For immunodetection, membranes were blocked with 5% milk in TTBS for 1 h at RT and then incubated OV with specific primary antibodies, suitably diluted in blocking solution (Supplementary Table 1). Membranes were washed three times in TTBS and incubated for 2 h at RT with the appropriate secondary antibodies conjugated with HRP (Supplementary Table 2). Finally, membranes were incubated with Crescendo Western HRP substrate (Milipore Burlington, MA, United States) and exposed using a ChemiDoc TM XRS + Imaging System (BioRad, Hercules, CA, United States).

**Quantitative Real-Time PCR**

Quantitative real-time PCR (qPCR) was used to assess gene expression. The RPLPO housekeeping gene served as the normalization control. RNA was isolated from skeletal muscle using Trizol (Invitrogen, Carlsbad, CA, United States), following the manufacturer’s instructions. DNase-treated RNA (1 μg) was retrotranscribed with Iscript cDNA synthesis Kit (Bio-Rad, Hercules, CA, United States) to produce complementary DNA (cDNA).

qPCR was performed using a CFX Connect Real-Time PCR Detection System (Bio-Rad, Hercules, CA, United States) with iTaq universal SYBR Green Supermix kit (Bio-Rad, Hercules, CA, United States) in 96-well reaction plates according to manufacturer’s instructions. Primers sequences can be found in Supplementary Table 3.

**Skeletal Muscle Histology and Immunochemistry**

The tibialis anterior (TA) muscle was carefully dissected, embedded in Tissue-Tec OCT^TM^ (Sakura, Torrance, CA, United States) and immediately frozen in liquid nitrogen cooled isopentane. Cryosections of TA muscle (12 μm) were obtained with a cryostat (Leica CM1850 UV-1-1; Leica, Wetzler, Germany) and stored at -80ºC until needed. For muscle fiber type composition, slides were washed with PBS and incubated for 1 h with blocking solution [10% (v/v) fetal bovine serum (FBS) in PBS]. Then, a cocktail of primary antibodies (described in Supplementary Table 1) diluted in blocking solution was prepared and incubated for 1 h at RT, as previously described (18). Slides were then washed with PBS 3 times for 5 min and incubated with secondary antibodies (Supplementary Table 2) for 1 h in darkness. For dystrophin immunostaining, slides were washed three times in PBS + 0.1% (v/v) Triton X100 for 8 min. Slides were incubated for 1 h with the blocking solution and then incubated with dystrophin antibody (Supplementary Table 1) diluted in blocking solution OV. Next, slides were washed 3 times for 5 minutes with PBS and incubated with the secondary antibody (Supplementary Table 2) for 2 h in darkness. Afterwards, slides were incubated with DAPI (Sigma-Aldrich, Spruce St, MO, United States) (1 μg/ml). For both methods, slides were washed 3 times for 5 min with PBS and mounted using glycerol 50% (v/v) under a coverslip. All steps were performed in a humidified chamber. Image acquisition was performed using a Stellaris Confocal Laser Scanning Microscope (Leica, Wetzler, Germany) (10X objective); complete reconstruction of the transversal section of the TA muscle was automatically generated by the Leica software. For TA myofiber type composition, a section of 2000 μm × 2000 μm, for each sample, including red and white areas, from the TA muscles was used for comparative analysis, which was performed with Fiji (ImageJ) by quantifying the number of the different types of myofibers. For dystrophin immunostaining, the whole muscle section was examined with Fiji (ImageJ) (19).

TUNEL staining in TA muscle was performed using the In Situ Cell Death Detection Kit with Fluorescein (Roche, ref. 11684795910) following the manufacturer's instructions. Briefly, muscle cryosections were fixed in 4% paraformaldehyde/PBS for 20 minutes at RT, washed three times in PBS for 10 minutes each, and permeabilized for 2 minutes at 4°C in ice-cold permeabilization solution (0.1% Triton X-100 + 0.1% NaCitrate in PBS). For labeling, samples were washed twice in PBS and incubated for 1 hour at 37°C with 50 microliters of freshly prepared TUNEL assay mix inside a humid chamber and in the dark. Samples were then washed in PBS and incubated for another 10 minutes with DAPI. Finally, samples were washed three times in PBS, mounted with glycerol, and observed under a fluorescence microscope (excitation wavelength 450-500 nm and detection wavelength at 515-565 nm). Image acquisition was performed using a Stellaris Confocal Laser Scanning Microscope, and analysis was conducted with Fiji (ImageJ) two section of 1000 μm × 1000 μm were examined and the mean of both sections is represented.

**Primary Satellite Cell Culture from Mouse Skeletal Muscle**

Satellite cells were isolated as previously described (20). Clean myofibers from extensor digitorum longus (EDL) muscle were plated in proliferation medium [59% (v/v) DMEM + Glutamax (Gibco, Waltham, MA, United States), 30% (v/v) FBS (Gibco, Waltham, MA, United States), 10% (v/v) horse serum (HS) (Gibco, Waltham, MA, United States), 1% (v/v) chicken embryo extract (CEE) (ThermoFisher, Waltham, MA, United States), 0.0001% (v/v) fibroblast growth factor (FGF) (Prepotech, Rocky Hill, NJ, United States), 1% (v/v) penicillin-streptomycin (PS) (Sigma-Aldrich, Spruce St, MO, United States)] in a Petri dish precoated with Matrigel (Sigma-Aldrich, Spruce St, MO, United States) [1:10 (v/v) in DMEM+Glutamax ] for 72 hours to allow satellite cells activation and migration. Finally, satellite cells were isolated and amplified in proliferation medium, and plated in differentiation medium [97.0% (v/v) DMEM + Glutamax (Gibco, Waltham, MA, United States], 1% PS (Sigma-Aldrich, Spruce St, MO, United States) and 2% HS).

For CoQ_10_ supplementation in cells, 0.53 mg of CoQ_10_ was mixed with 50 μl of HPLC Grade ethanol and heated at 40ºC until dissolved. This solution was added to 0.5 ml of HS previously heated at 37ºC. The aliquot was added to a 50 ml medium for a final concentration of 10 μM.

**Immunostaining of Satellite Cells**

Satellite cells were plated in a 96-well plate previously coated with Matrigel [1:10 (v/v) in DMEM + Glutamax (Gibco, Waltham,MA, United States)] for the assays. Cells were fixed with 100 μl of 4% (w/v) paraformaldehyde per well, and the plate was transferred to an orbital shaker for 8 min. Cells were then washed 3 times with PBS for 5 min and permeabilized with 0.1% (v/v) Triton X-100/PBS shaking for 15 min. Cells were incubated with blocking solution (5% [v/v] FBS/PBS) shaking for 1 h. Myosin Heavy Chain (MyHC) antibody diluted in blocking solution (1:100) (Supplementary Table 1) was incubated OV shaking at 4°C. Cells were washed 3 times with PBS for 5 min and the secondary antibody (Supplementary Table 2) was incubated shaking for 2 h in darkness. Cells were washed with PBS and incubated with DAPI (Sigma-Aldrich, Spruce St, MO, United States) (1 μg/ml) shaking for 10 min. All steps were performed at RT. Images were acquired on a Fluorescence Microscopy Zeiss Axio Imager M2 (Carl Zeiss Meditec AG, Oberkochen, Germany) (10X objective). MyHC area was calculated using a high throughput image analysis R script (21). Five images per well were used to calculate the mean MyHC area, whereas five wells were used for each cell line and time point.

To evaluate mitochondrial morphology, myogenic cells were cultured on coverslips placed in 12-well cell culture plates containing proliferation medium. The cells were stained with MitoTracker Deep Red (Thermo Fisher Scientific M22426) [200 nM] for 45 minutes at 37°C and 5% CO2. Following staining, the cells were fixed with 750 μl of a 1:1 (v/v) acetone and methanol solution for 10 minutes at 4°C. The fixed cells were then washed three times with PBS and mounted on slides using mounting media. Images were captured using a Leica SPE Confocal Microscope (Leica, Wetzlar, Germany) with a 63X objective. To quantify mitochondrial parameters, total mitochondrial volume was determined using Trainable Weka Segmentation (a Fiji plugin), followed by image binarization.

**Assessment of In Vivo Skeletal Muscle Regeneration**

Barium chloride (BaCl_2_) [50 μL of 1.2% w/v in water] was injected into the right TA of mice to produce skeletal muscle damage and induce satellite cell-mediated muscle regeneration (22). Mice were anaesthetized (ketamine [65 mg/kg body weight], xylazine [13 mg/kg body weight] and acepromazine [2 mg/kg body weight]) prior to injection and were kept warm during recovery and before being returned to their cage. The skin was shaved over the TA muscle. Trained personnel conducted daily observations of the animals to monitor their recovery. Next, 14 days post injection (dpi) mice were sacrificed, and the TA was carefully dissected, frozen and immunolabelled as described above. Dystrophin immunostaining and DAPI were used to determined nuclei position in transversal section of myofibers during regeneration. Central myonuclei quantification was performed with Fiji (ImageJ) (included in Supplementary Table 4).

**Assessment of Mitochondrial Respiration in Satellite Cell Derived Myotubes**

Agilent Seahorse XFe24 (Agilent, Santa Clara, CA, United States) microplate-based oxygen consumption technology was used to analyse respiration in myotubes differentiated from satellite cells. Previously, the XFe24 microplate was precoated with Matrigel. Then, freshly isolated satellite cells were seeded at an optical density of 30,000 cells and cultured under differentiation medium for 72 h. Oxygen consumption rate (OCR) was measured in Agilent Seahorse XF base medium supplemented with 2 mM D-glucose, 1 mM sodium pyruvate and 2 mM L-glutamine and pH was adjusted to 7.4. Substrates and inhibitors were sequentially loaded in the cartridge as follows: oligomycin [4 μM final], carbonyl cyanide-ptrifluoromethoxy phenylhydrazone (FCCP) [1.0 μM final], antimycin A [5 μM final] and rotenone [1 μM final]. Respiratory parameters for OCR assays were calculated as previously described(23). After measurements were obtained, cells were lysed in RIPA buffer for protein quantification. RC DC Protein Assay kit (Bio-Rad 5000122) was used to quantify protein content and albumin (Bio-Rad 5000007) was used as the standard for normalization.

**Assessment of Respiration in Isolated Mitochondria from Skeletal Muscle**

To assess mitochondrial metabolism and bioenergetics, mitochondria were isolated from skeletal muscle (including the tibialis anterior, extensor digitorum longus, gastrocnemius, soleus, quadriceps, peroneus longus, peroneus brevis, and the plantaris muscles) through serial centrifugations, and 10 μg of functional and living mitochondria were seeded per well as previously described (14). Assays were conducted using an Agilent Seahorse XFe24 microplate-based oxygen consumption system. The Coupling Assay (CA) examines mitochondrial states in the presence of pyruvate and malate as substrates. The β-oxidation of fatty acids assay (BOX) was carried out in the presence of palmitoyl-l-carnitine and malate. Substrates and inhibitors were sequentially added as follows: ADP [5 mM final] for CA and [4mM final] for BOX, Oligomycin [3.16 μM final], FCCP [6 μM final] and antimycin A plus rotenone [4 and 5 μM final] for CA or antimycin A plus rotenone [4 and 2 μM final], for BOX.

**Statistics**

Data were expressed as mean ± SD. Data analyses were performed with GraphPad Prism 9.0 software. When a comparison between two groups was performed, an unpaired two-tailed t-test was used. When more than two groups were studied, a one way (1 parameter studied) or two-way ANOVA (2 or more parameters studied) with Tukey’s multiple comparison post-hoc tests was applied. p-values < 0.05 were considered statistically significant.

**References for Supplementary methods section:**

1. Vázquez-Fonseca L, Schaefer J, Navas-Enamorado I, Santos-Ocaña C, Hernández-Camacho JD, Guerra I, et al. ADCK2 haploinsufficiency reduces mitochondrial lipid oxidation and causes myopathy associated with CoQ deficiency. J Clin Med. 2019 Sep 2;8(9):1374.

2. Rötig A, Appelkvist EL, Geromel V, Chretien D, Kadhom N, Edery P, et al. Quinone-responsive multiple respiratory-chain dysfunction due to widespread coenzyme Q10 deficiency. Lancet. 2000 Jul 29;356(9227):391–5.

3. Montini G, Malaventura C, Salviati L. Early coenzyme Q10 supplementation in primary coenzyme Q10 deficiency. N Engl J Med. 2008 Jun 26;358(26):2849–50.

4. Navas P, Cascajo MV, Alcázar-Fabra M, Hernández-Camacho JD, Sánchez-Cuesta A, Rodríguez ABC, et al. Secondary CoQ10 deficiency, bioenergetics unbalance in disease and aging. Biofactors. 2021 Jul;47(4):551–69.

5. Aartsma-Rus A, van Putten M. Assessing functional performance in the mdx mouse model. J Vis Exp [Internet]. 2014 Mar 27;(85). Available from: http://dx.doi.org/10.3791/51303-v

6. Deacon RMJ. Measuring motor coordination in mice. J Vis Exp. 2013 May 29;(75):e2609.

7. Fernández-Calleja JMS, Konstanti P, Swarts HJM, Bouwman LMS, Garcia-Campayo V, Billecke N, et al. Non-invasive continuous real-time in vivo analysis of microbial hydrogen production shows adaptation to fermentable carbohydrates in mice. Sci Rep. 2018 Oct 18;8(1):15351.

8. Tajbakhsh S. Skeletal muscle stem cells in developmental versus regenerative myogenesis. J Intern Med. 2009 Oct;266(4):372–89.

9. Agarwal M, Sharma A, Kumar P, Kumar A, Bharadwaj A, Saini M, et al. Myosin heavy chain-embryonic regulates skeletal muscle differentiation during mammalian development. Development [Internet]. 2020 Apr 1;147(7). Available from: http://dx.doi.org/10.1242/dev.184507

10. Gordillo M, Evans T, Gouon-Evans V. Orchestrating liver development. Development. 2015 Jun 15;142(12):2094–108.

11. Carpenter B, Lin Y, Stoll S, Raffai RL, McCuskey R, Wang R. VEGF is crucial for the hepatic vascular development required for lipoprotein uptake. Development. 2005 Jul 15;132(14):3293–303.

12. Di Bella DJ, Habibi E, Stickels RR, Scalia G, Brown J, Yadollahpour P, et al. Molecular logic of cellular diversification in the mouse cerebral cortex. Nature. 2021 Jul 22;595(7868):554–9.

13. Fernández-Ayala DJM, Guerra I, Jiménez-Gancedo S, Cascajo MV, Gavilán A, Dimauro S, et al. Survival transcriptome in the coenzyme Q10 deficiency syndrome is acquired by epigenetic modifications: a modelling study for human coenzyme Q10 deficiencies. BMJ Open. 2013 Mar 25;3(3):e002524.

14. Hernández-Camacho JD, Vicente-García C, Sánchez-Cuesta A, Fernandez-Ayala DJM, Carvajal JJ, Navas P. Isolation of mitochondria from mouse skeletal muscle for respirometric assays. J Vis Exp. 2022 Feb 10;(180). Available from: http://dx.doi.org/10.3791/63336-v

15. Rodríguez-Aguilera JC, Cortés AB, Fernández-Ayala DJM, Navas P. Biochemical assessment of coenzyme Q10 deficiency. J Clin Med. 2017 Mar 5;6(3):27.

16. Gilda JE, Gomes AV. Stain-Free total protein staining is a superior loading control to β-actin for Western blots. Anal Biochem. 2013 Sep 15;440(2):186–8.

17. Jha P, Wang X, Auwerx J. Analysis of mitochondrial respiratory chain supercomplexes using blue native polyacrylamide gel electrophoresis (BN-PAGE). Curr Protoc Mouse Biol. 2016 Mar 1;6(1):1–14.

18. Bloemberg D, Quadrilatero J. Rapid determination of myosin heavy chain expression in rat, mouse, and human skeletal muscle using multicolor immunofluorescence analysis. PLoS One. 2012 Apr 18;7(4):e35273.

19. Schindelin J, Arganda-Carreras I, Frise E, Kaynig V, Longair M, Pietzsch T, et al. Fiji: an open-source platform for biological-image analysis. Nat Methods. 2012 Jun 28;9(7):676–82.

20. Moyle LA, Zammit PS. Isolation, culture and immunostaining of skeletal muscle fibres to study myogenic progression in satellite cells. Methods Mol Biol. 2014;1210:63–78.

21. Banerji CRS, Panamarova M, Pruller J, Figeac N, Hebaishi H, Fidanis E, et al. Dynamic transcriptomic analysis reveals suppression of PGC1α/ERRα drives perturbed myogenesis in facioscapulohumeral muscular dystrophy. Hum Mol Genet. 2019 Apr 15;28(8):1244–59.

22. Morton AB, Norton CE, Jacobsen NL, Fernando CA, Cornelison DDW, Segal SS. Barium chloride injures myofibers through calcium-induced proteolysis with fragmentation of motor nerves and microvessels. Skelet Muscle. 2019 Nov 6;9(1):27.

23. Divakaruni AS, Paradyse A, Ferrick DA, Murphy AN, Jastroch M. Analysis and interpretation of microplate-based oxygen consumption and pH data. Methods Enzymol. 2014;547:309–54.
