# Supplementary material for: Prenatal and progressive coenzyme Q10 administration to mitigate muscle dysfunction in mitochondrial disease
Source: J Cachexia Sarcopenia Muscle. 2024 Oct 2;15(6):2402–16. doi: 10.1002/jcsm.13574 (PMC11634497; doi:10.1002/jcsm.13574)
Supplement: Supplementary file 3 — Table S1. Primary antibodies. Table S2. Secondary antibodies. Table S3. Primers for qPCR. Table S4. Image analysis pipeline used in Fiji. Table S5. Regression lines for exercise tests. [file JCSM-15-2402-s001.docx]

Title: Prenatal and progressive Coenzyme Q_10_ administration to mitigate muscle dysfunction in mitochondrial disease.

Juan Diego Hernández-Camacho^1,2^*, Cristina Vicente-García^1^, Lorena Ardila-García^1^, Ana Padilla-Campos^1^, Guillermo López-Lluch^1,2^, Carlos Santos-Ocaña^1,2^, Peter S. Zammit^3^, Jaime J. Carvajal^1^, Plácido Navas^1,2^ and Daniel J. M. Fernández-Ayala^1,2^*.

1 Centro Andaluz de Biología del Desarrollo-CSIC, Universidad Pablo de Olavide, ES-41013 Seville, Spain

2 CIBERER, Instituto de Salud Carlos III, Madrid, Spain.

3 King's College London, Randall Centre for Cell and Molecular Biophysics, London, SE1 1UL, UK.

*Correspondence to: jdhercam@alu.upo.es and dmorfer@upo.es

**Supplementary Table 1. Primary antibodies**

| **Antigen** | **Mono/Polyclonal** | **Host species** | **Dilution** | **Company** | **Reference** | **Application** |
| --- | --- | --- | --- | --- | --- | --- |
| MHC | Monoclonal | Mouse | 1:300 | DSHB | MF20-c | IHC |
| Myosin heavy chain Type IIB | Monoclonal | Mouse | 1:100 | DSHB | BF-F3 | IHC |
| Myosin heavy chain Type IIA | Monoclonal | Mouse | 1:600 | DSHB | SC-71 | IHC |
| Dystrophin | Monoclonal | Mouse | 1:100 | DSHB | MANDRA4(5H7) | IHC |
| TIMM23 | Polyclonal | Rabbit | 1:1000 | Abcam | Ab230253 | WB |
| VDAC | Polyclonal | Rabbit | 1:1000 | Abcam | Ab15895 | WB |
| TFAM | Polyclonal | Goat | 1:1000 | SC | Sc-23588 | WB |
| MASPIN | Monoclonal | Mouse | 1:500 | SC | Sc-271694 | WB |
| TUBULIN | Monoclonal | Mouse | 1:500 | SC | Sc-398103 | WB |
| NDUFA9 | Monoclonal | Mouse | 1:1000 | Abcam | Ab14713 | WB |
| SDHA | Monoclonal | Mouse | 1:1000 | Invitrogen | 459200 | WB |
| UQCRC2 | Polyclonal | Rabbit | 1:1000 | Abcam | Ab14745 | WB |
| MT-CO1 | Monoclonal | Mouse | 1:2000 | Invitrogen | 459600 | WB |
| MT-ATP6 | Monoclonal | Mouse | 1:1000 | Abcam | Ab14748 | WB |
| Mfn2 | Polyclonal | Rabbit | 1:1000 | Abcam | Ab50838 | WB |
| Pink1 | Monoclonal | Mouse | 1:1000 | Abcam | Ab75487 | WB |

Developmental Studies Hybridoma Bank (DSHB), Santa Cruz (SC), Western blot (WB), Immunohistochemistry (IHC).

**Supplementary Table 2. Secondary antibodies**

| **Antigen** | **Host species** | **Dilution** | **Company** | **Reference** | **Target** | **Conjugate** |
| --- | --- | --- | --- | --- | --- | --- |
| Anti-Goat | Rabbit | 1:5000 | Sigma | 401504 | IgG | HRP |
| Anti-Rabbit | Goat | 1:5000 | CS | 7074 | IgG | HRP |
| Anti-Mouse | Goat | 1:5000 | JL | 115-035-006 | IgG | HRP |
| Anti-Mouse IgG (H+L) | Goat | 1:1000 | Thermo | A-11001 | IgG | Alexa 488 |
| Anti-Mouse IgG1 | Goat | 1:500 | Thermo | A-21121 | IgG1 | Alexa 488 |
| Anti-Mouse IgM | Goat | 1:500 | Thermo | A-21426 | IgM  (Heavy chain) | Alexa 555 |

Cell Signaling (CS), Jackson Laboratory (JL).

**Supplementary Table 3. Primers for qPCR.**

| **Target**  **genes** | **Forward (5’-3’)** | **Reverse (5’-3’)** |
| --- | --- | --- |
| *Rplpo* | AGA TTC GGG ATA TGC TGT TGG C | TCG GGT CCT AGA CCA GTG TTC |
| *Adck2* | GGG TCT CTT TCG GAT CTG GGG CAG | TGG TGG GTC AGA AGT GGG TGT GTC |
| *Ttr* | TTG CCT CGC TGG ACTG GTA | TTA CAG CCA CGT CTA CAG CAG |
| *Apoa2* | TGG TCG CAG TGC TGG TAA C | TTT GCC ATA TTC AGT CAT GCT CT |

**Supplementary Table 4. Image analysis pipeline used in Fiji.**

| Creation of a Green and Blue Channels Mask, accomplished through the following steps: |
| --- |
| - Image › Color › Split Channels. Select the green channel. |
| - Process › Enhance Local Contrast (CLAHE) |
| - Process › Subtract Background (50 pixels) |
| - Process › Smooth (X3) |
| - Image › Adjust › Threshold |
| - MorphoLibJ › Morphological Filters › Closing + Disk |
| - Edit › Invert |
| - Analyze › Analyse Particles |
| - Subsequently, the Region of Interest (ROI) was loaded in the original color image. The image type was changed to RGB Color. Using the drawing tool from the Fiji toolbar, the myofiber border was painted in white, while the myofibers' sharp edges were painted in red (Manual curating). The image was then transformed to 8-bit (Image › Type › RGB Color) |
| - Particles were selected separately and pasted onto the original image (mask). The mask for the green channel was considered complete. The next steps were performed for the green channel mask: open green channel mask and Analyze › Analyse Particles |
| - For Blue channel mask › Process › Filters › Gaussian Blur. |
| - Process › Binary › Make Binary |
| Select the central myonuclei: |
| - The ROI generated on the green channel mask was then loaded into the blue channel mask. Edit › Clear Outside was used to remove peripheral nuclei. |
| - The following analyses were subsequently conducted to examine central nuclei within the particles generated in the green mask: Analyze › Analyze Particles and Analyze › Summarize |

**Supplementary Table 5. Regression lines for exercise tests.**

| **Parameter: Weights test** | | |
| --- | --- | --- |
| Group | Graph equation | R squared on the graph |
| *Adck2^+/+^* | y = -0.0034x + 0.4148 | R² = 0.1411 |
| *Adck2^+/+^* +CoQ_10_ | y = -0.0036x + 0.441 | R² = 0.1626 |
| *Adck2^+/-^* | y = -0.0037x + 0.3699 | R² = 0.1751 |
| *Adck2^+/-^* +CoQ_10_ | y = -0.0013x + 0.3969 | R² = 0.0136 |
| **Parameter: Grip strength two limbs** | | |
| Group | Graph equation | R squared on the graph |
| *Adck2^+/+^* | y = -0.00016x + 0.02909 | R² = 0.07622 |
| *Adck2^+/+^* +CoQ_10_ | y = -0.00001x + 0.02540 | R² = 0.00066 |
| *Adck2^+/-^* | y = -0.00033x + 0.02710 | R² = 0.32095 |
| *Adck2^+/-^* +CoQ_10_ | y = -0.00005x + 0.02665 | R² = 0.00995 |
| **Parameter: Grip strength four limbs** | | |
| Group | Graph equation | R squared on the graph |
| *Adck2^+/+^* | y = -0.00028x + 0.04248 | R² = 0.12637 |
| *Adck2^+/+^* +CoQ_10_ | y = 0.00001x + 0.03788 | R² = 0.00015 |
| *Adck2^+/-^* | y = -0.00064x + 0.04482 | R² = 0.55984 |
| *Adck2^+/-^* +CoQ_10_ | y = -0.00004x + 0.03984 | R² = 0.00330 |
