# Supplementary material for: Prenatal and progressive coenzyme Q10 administration to mitigate muscle dysfunction in mitochondrial disease
Source: J Cachexia Sarcopenia Muscle. 2024 Oct 2;15(6):2402–16. doi: 10.1002/jcsm.13574 (PMC11634497; doi:10.1002/jcsm.13574)
Supplement: Supplementary file 4 — Figure S1. Impact of CoQ10 administration in different tissues. A. CoQ10 oxidation rate. Reduced and oxidized forms of CoQ10 in water were measured by HPLC at different time points during a 9‐day period. B. CoQ9, CoQ10 levels and ratio CoQ9/CoQ10 on plasma, spleen and liver from young mice. (Adck2 +/+ N = 3, Adck2 +/+ CoQ10 N = 3, Adck2 +/− N = 3, Adck2 +/− CoQ10 N = 3). C. Representative RNA integrity verification by electrophoresis in an agarose 1% (w/v) gel. D. Representative Stain‐Free gel image used as loading control in Western blotting assays. E. Protein markers of mitochondrial mass and skeletal muscle structural organization in skeletal muscle from 17 dpc embryos. (Adck2 +/+ N = 3, Adck2 +/− N = 3). dpc: days post‐coitum. Data represent the mean ± SD. One‐way ANOVA test was applied. *p < 0.05; **p < 0.01; ***p < 0.001; ****p < 0.0001. Figure S2. Impact of Adck2 ablation on the transcriptomic profile of skeletal muscle development. A. Apoptosis pathway genes expression in skeletal muscle from embryos at 17 dpc with/without CoQ10 prenatal administration. Activated gene expression is represented in green and repressed gene expression, in red (comparisons represented: Adck2 +/− vs Adck2 +/+ , Adck2 +/− + CoQ10 vs Adck2 +/− and Adck2 +/− + CoQ10 vs Adck2 +/+ ). N = 3 per group. B. Protein Turnover pathway genes expression in skeletal muscle from embryos at 17 dpc on basal conditions and with CoQ10 prenatal administration. Activated gene expression is represented in green and repressed gene expression, in red (comparisons represented: Adck2 +/− vs Adck2 +/+ , Adck2 +/− + CoQ10 vs Adck2 +/− and Adck2 +/− + CoQ10 vs Adck2 +/+ ). N = 3 per group. C. Mitochondrial biogenesis pathway genes expression in skeletal muscle from embryos at 17 dpc with/without CoQ10 prenatal administration. Activated gene expression is represented in green and repressed gene expression, in red (comparisons represented: Adck2 +/− vs Adck2 +/+ , Adck2 +/− + CoQ10 vs Adck2 +/− and Adck2 +/− + [file JCSM-15-2402-s004.pdf]

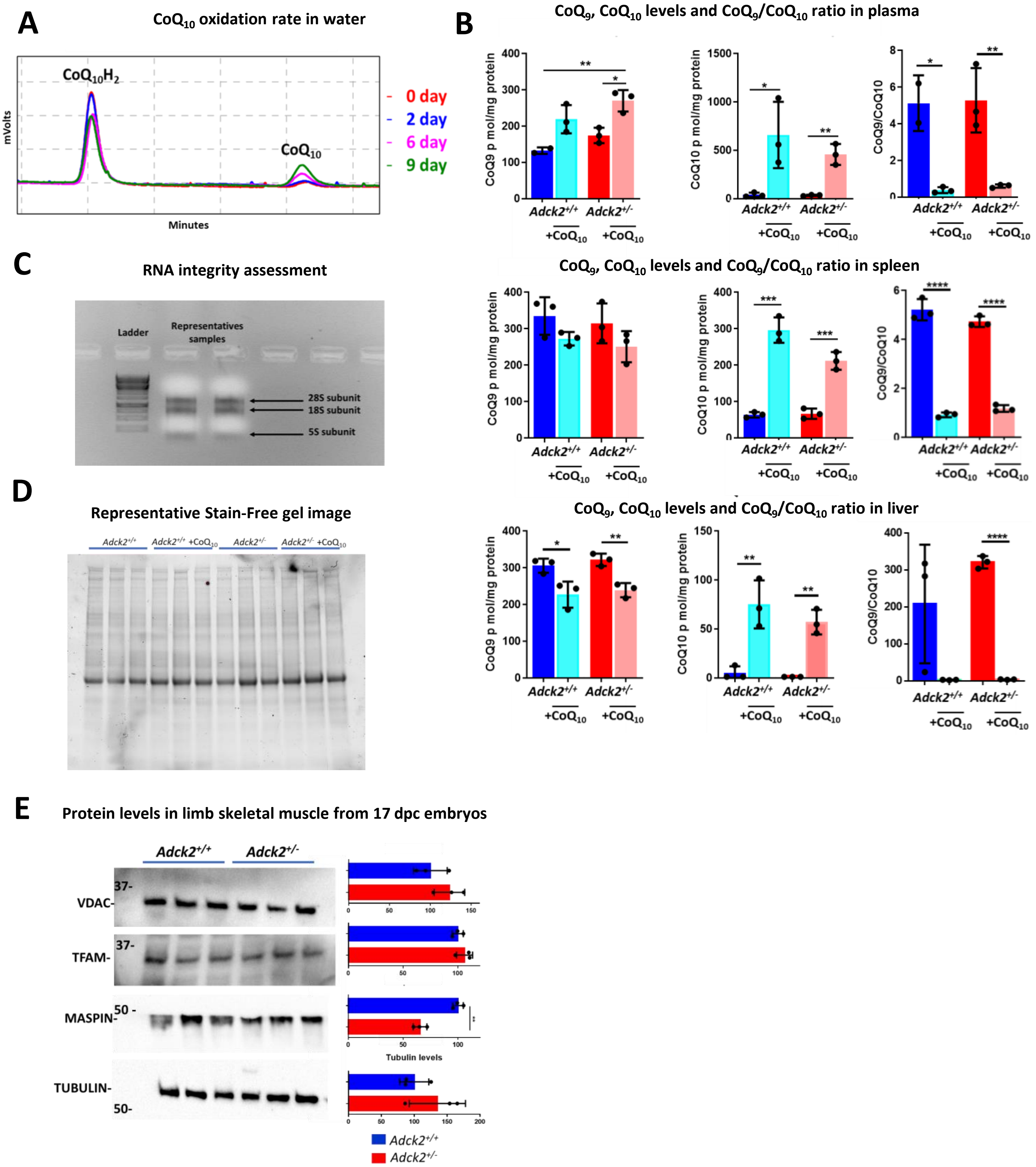

Figure Suppl 1

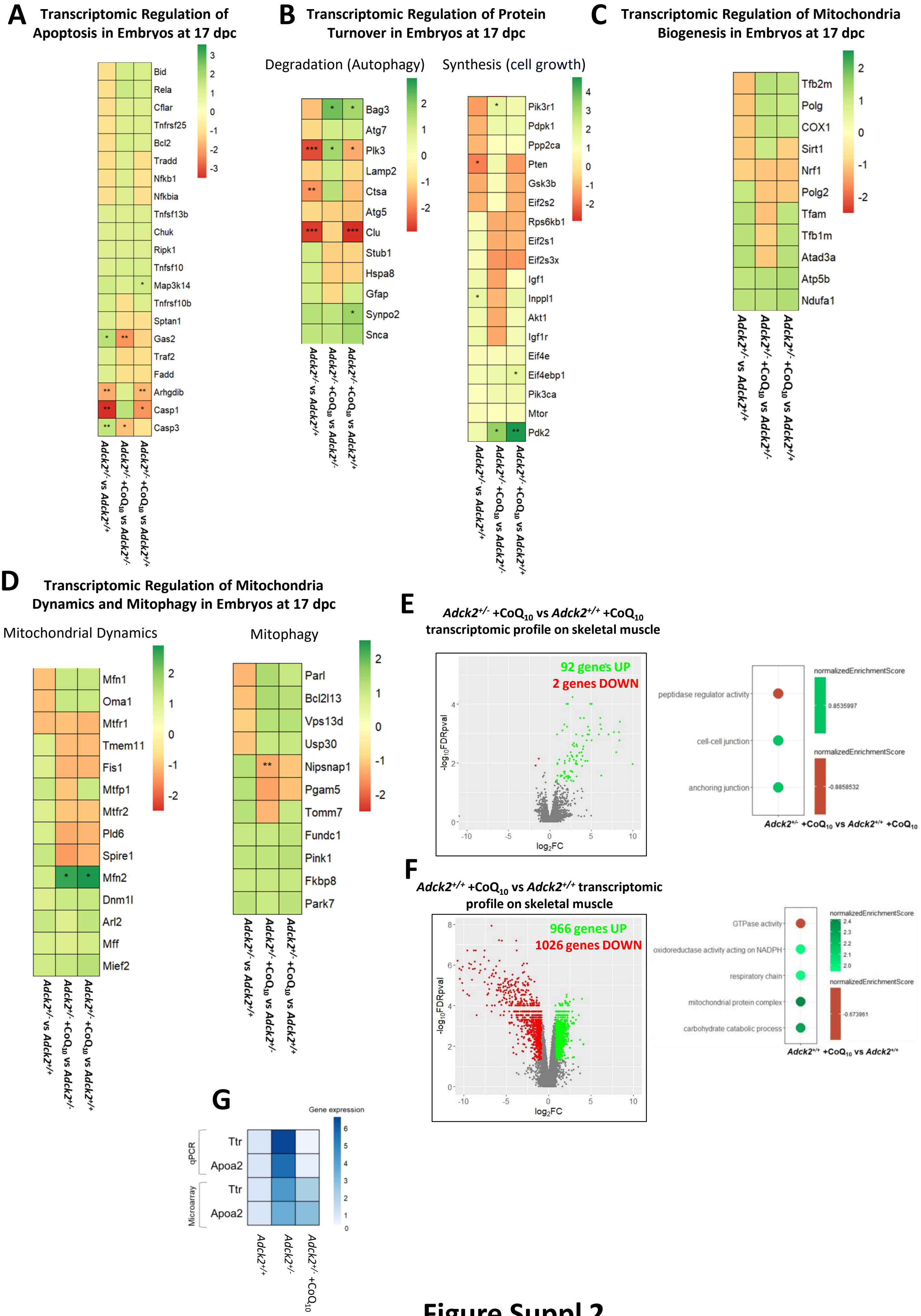

Figure Suppl 2

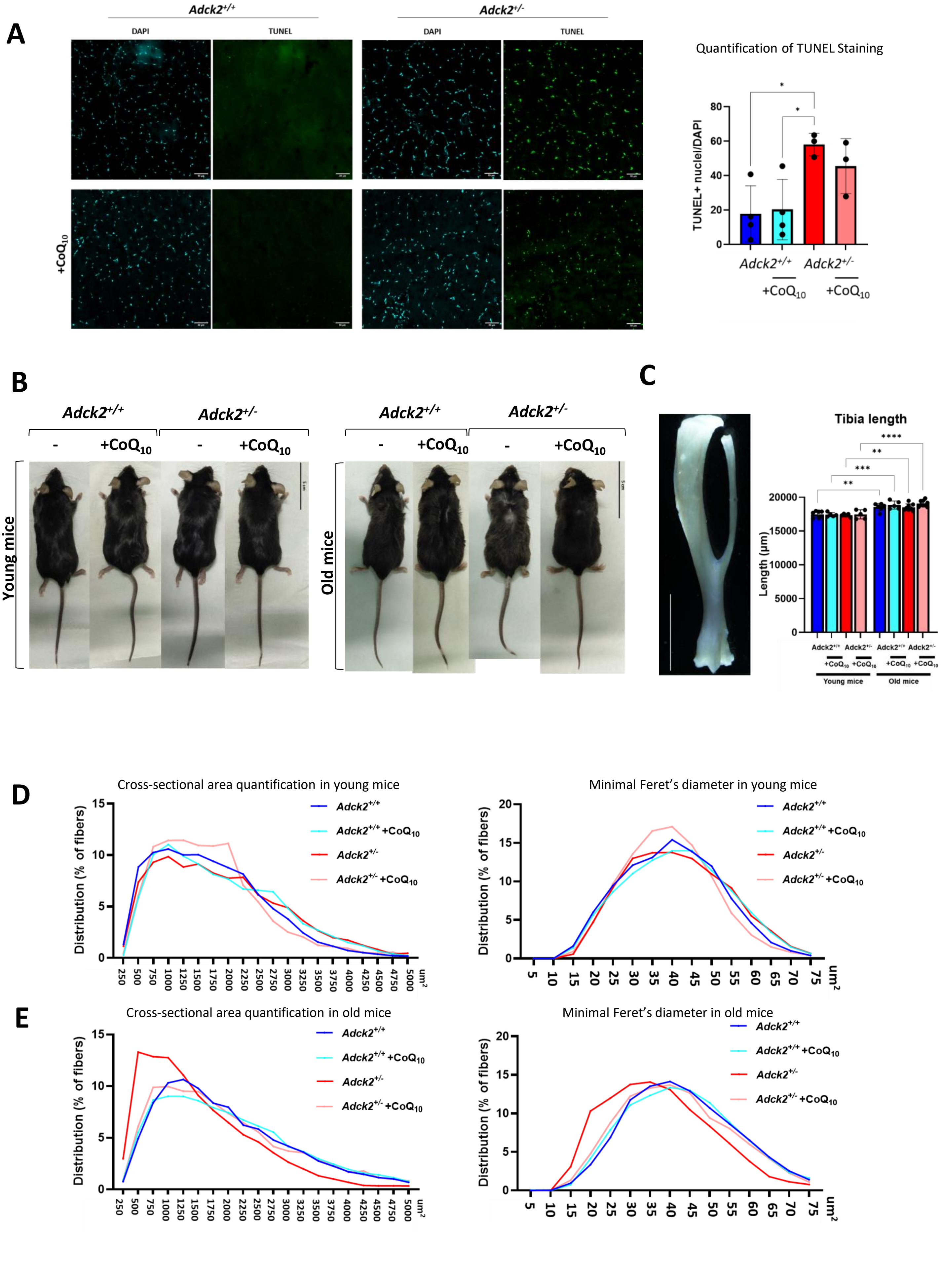

Figure Suppl 3

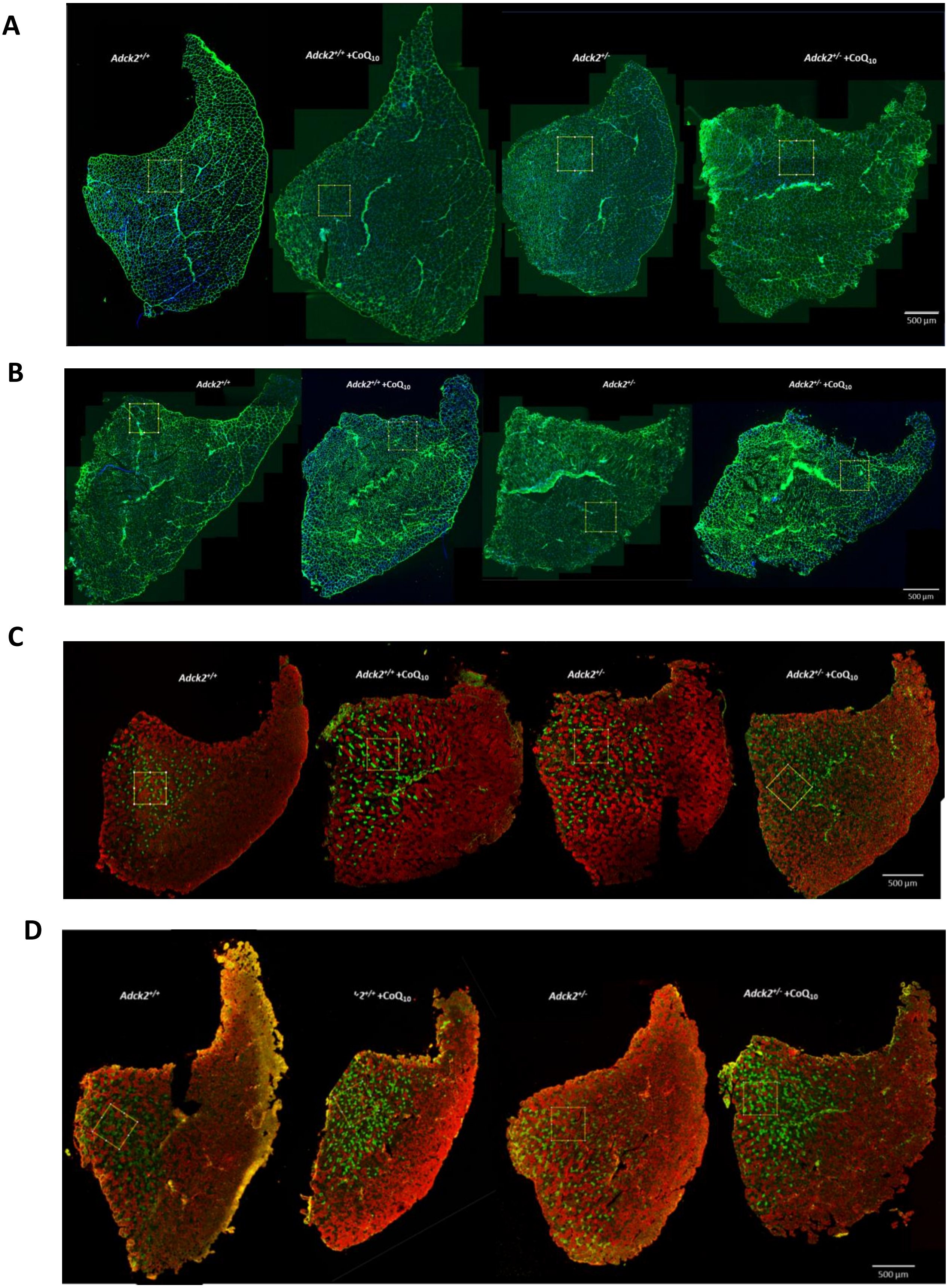

Figure Suppl 4

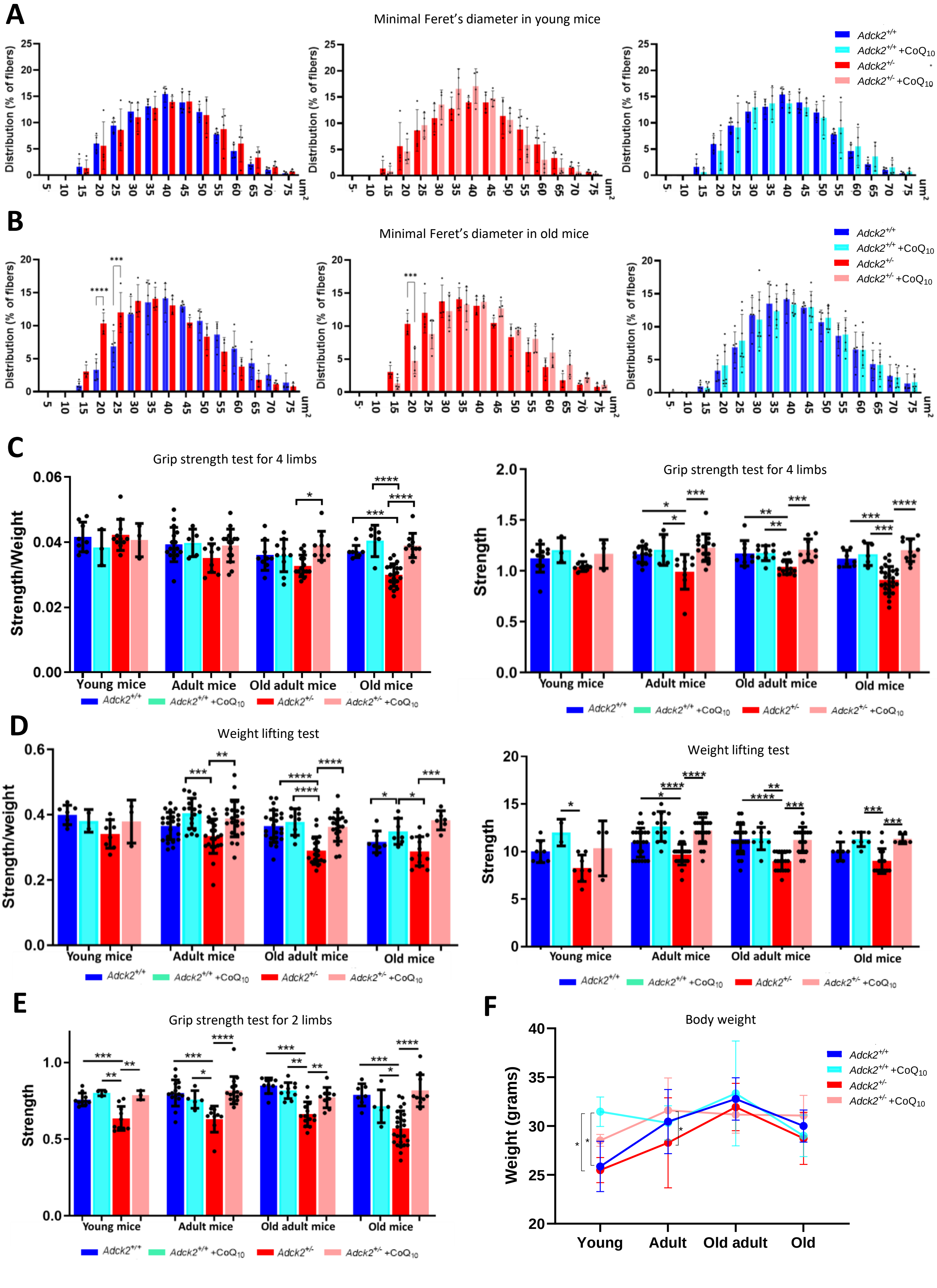

Figure Suppl 5

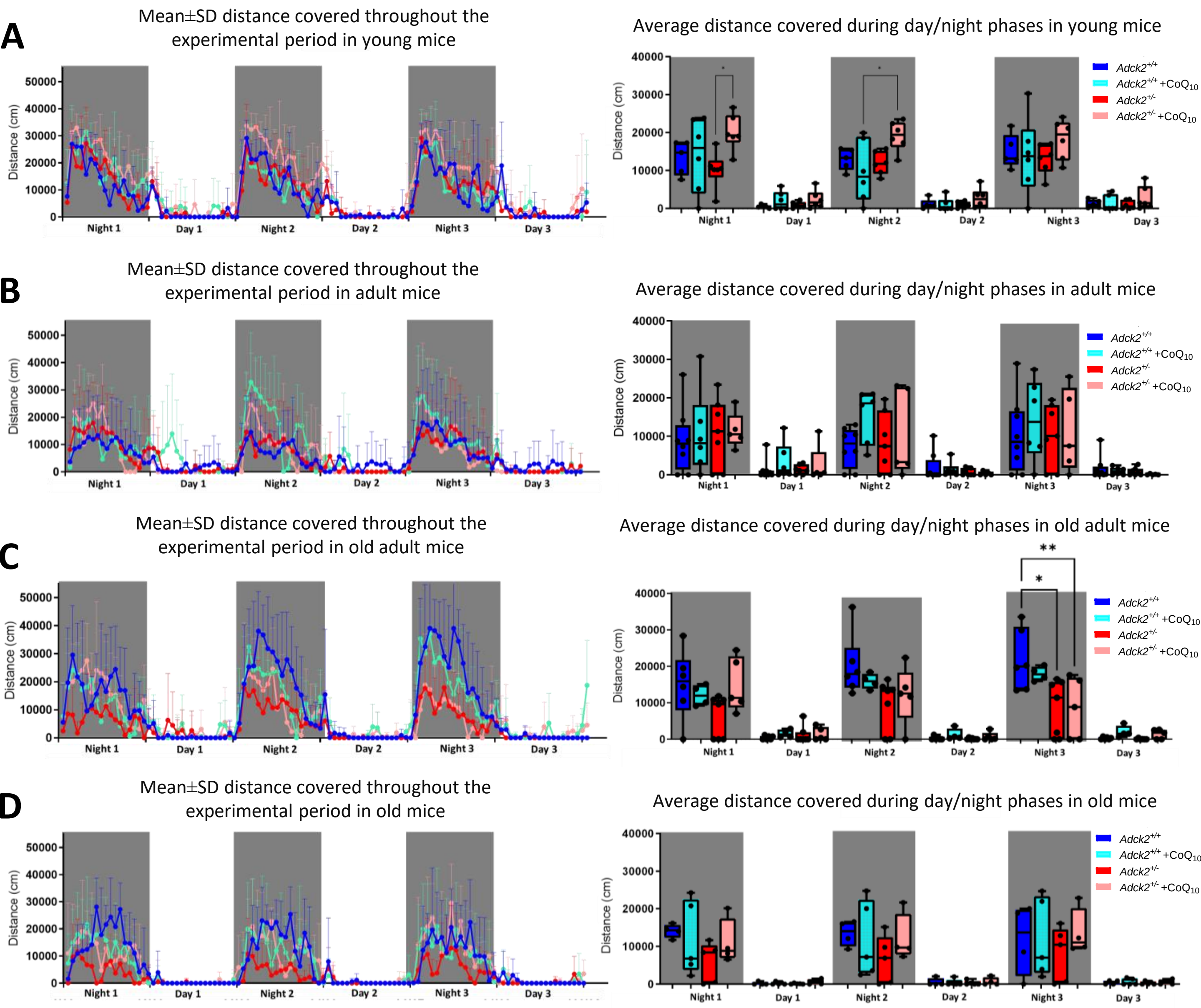

Figure Suppl 6

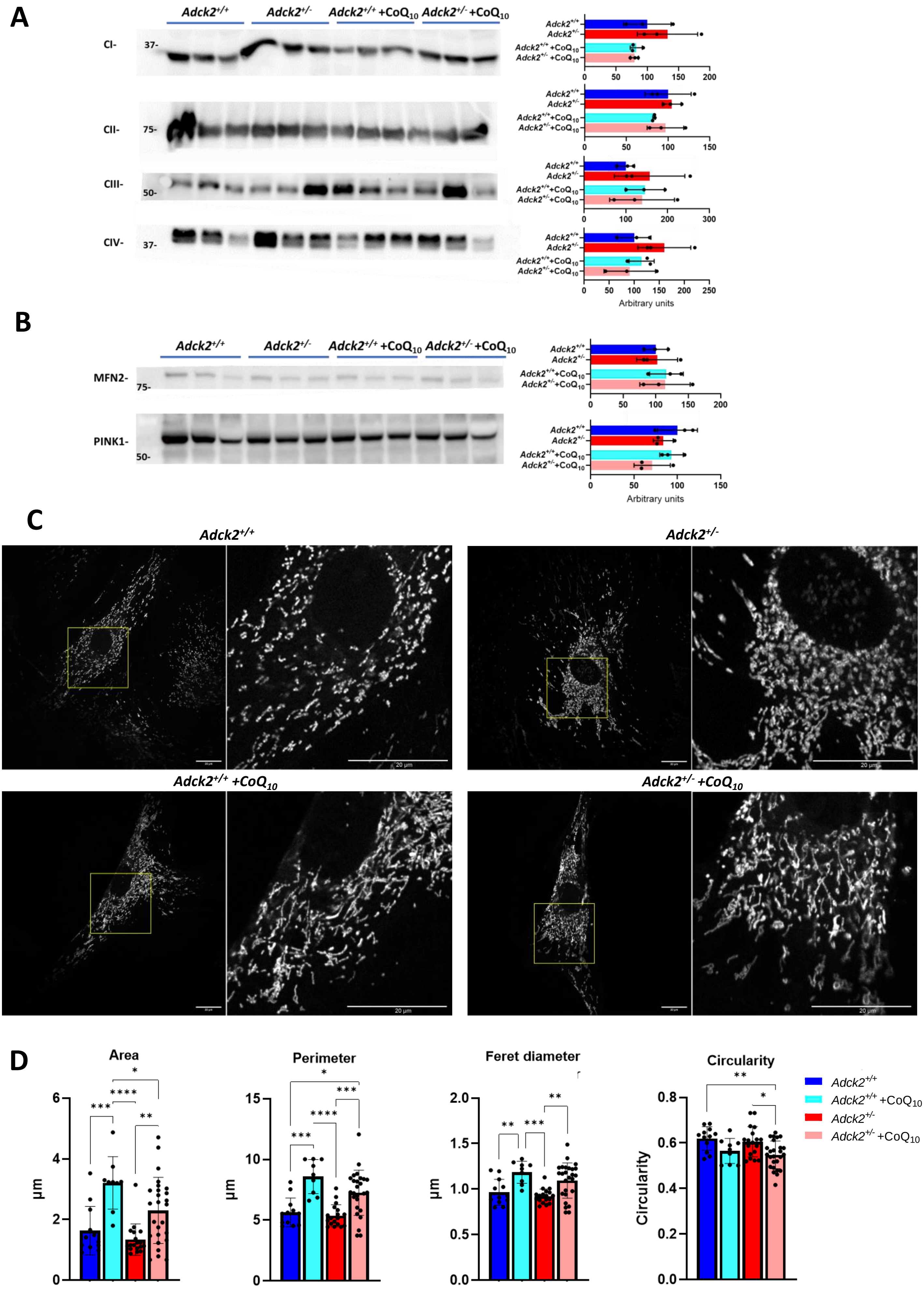

Figure Suppl 7

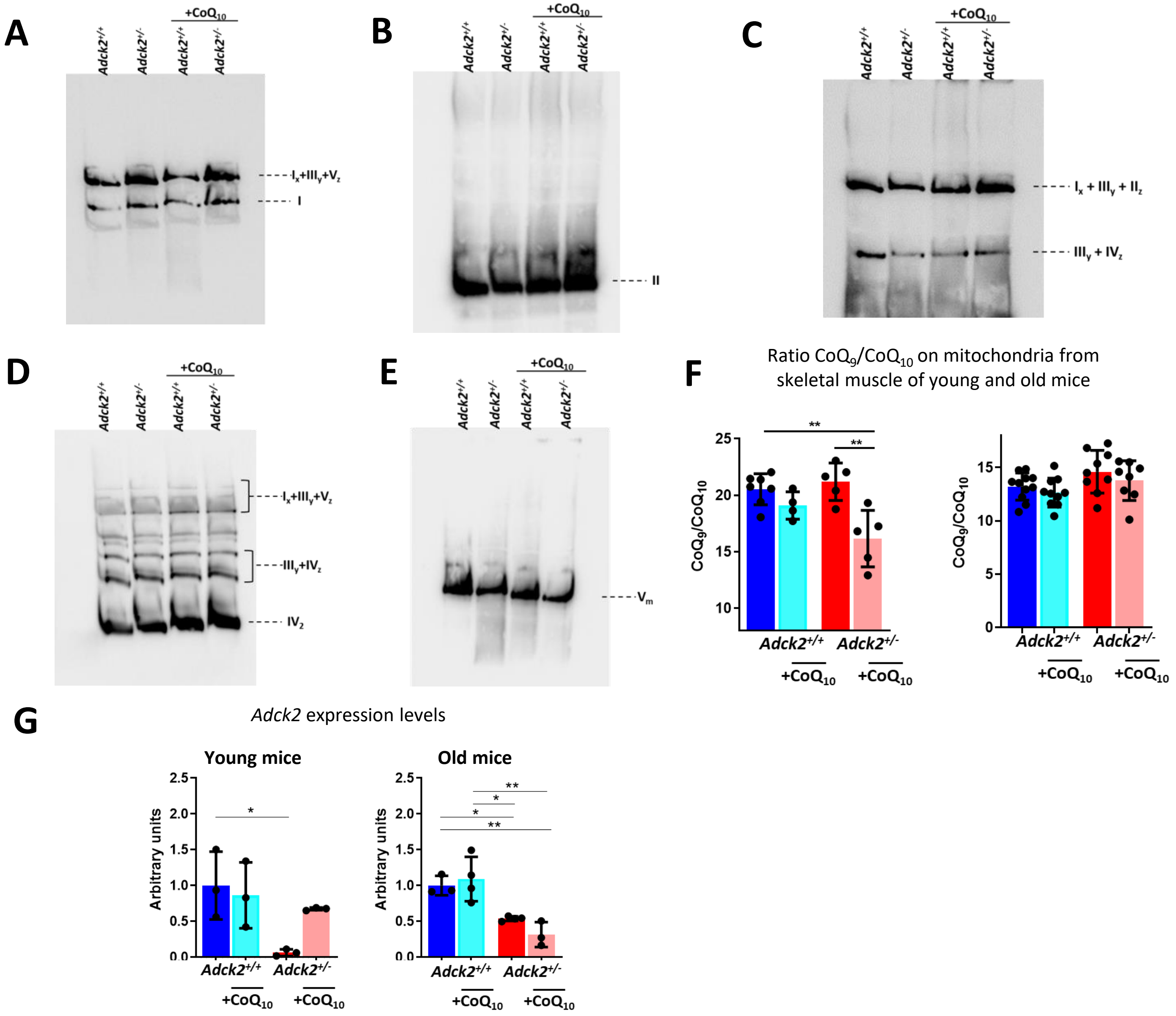

Figure Suppl 8

A

Young mice

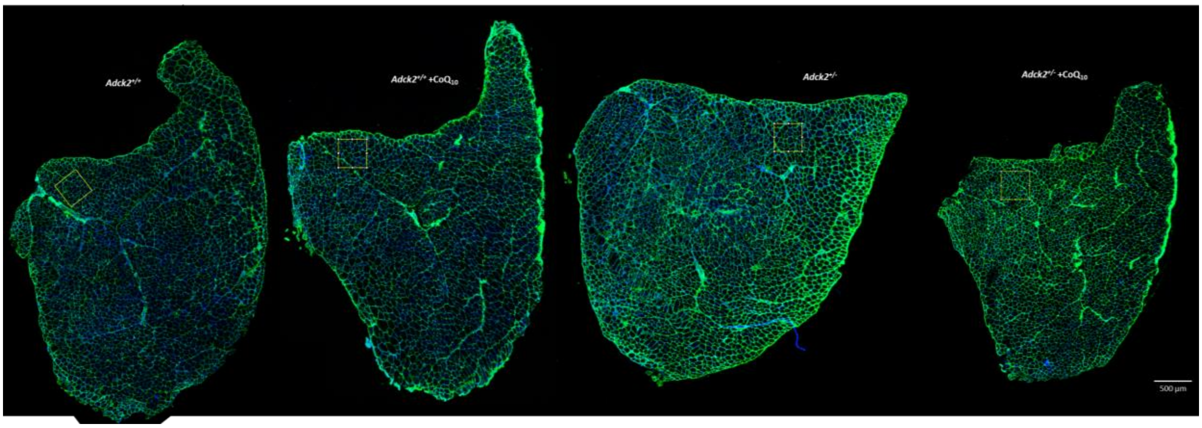

B

Old mice

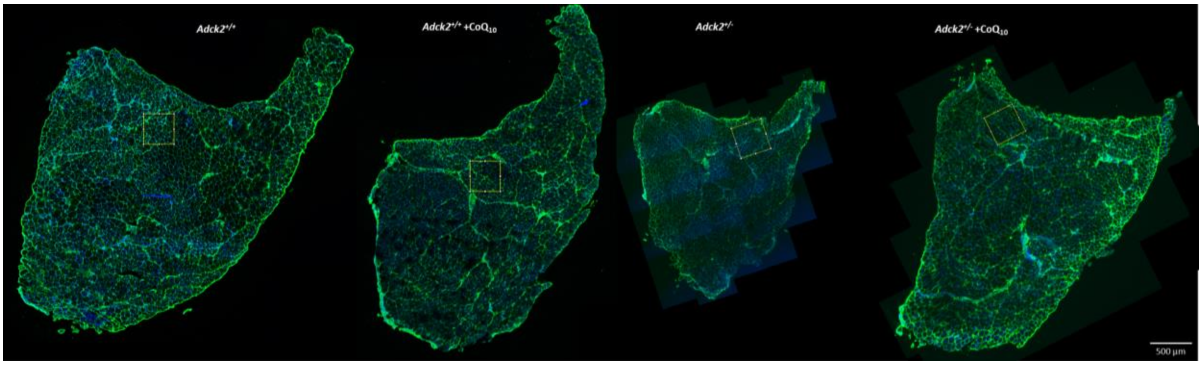

Figure Suppl 9
